# Single‐cell RNA sequencing reveals localized tumour ablation and intratumoural immunostimulant delivery potentiate T cell mediated tumour killing

**DOI:** 10.1002/ctm2.937

**Published:** 2022-07-08

**Authors:** Ashley R. Hoover, Kaili Liu, Christa I. DeVette, Jason R. Krawic, Alexandra D. Medcalf, Connor L. West, Tomas Hode, Samuel S.K. Lam, Alana L. Welm, Xiao‐Hong Sun, William H. Hildebrand, Wei R. Chen

**Affiliations:** ^1^ Stephenson School of Biomedical Engineering University of Oklahoma Norman Oklahoma USA; ^2^ Arthritis & Clinical Immunology Research Program Oklahoma Medical Research Foundation Oklahoma City Oklahoma USA; ^3^ Department of Microbiology and Immunology University of Oklahoma Health Sciences Center Oklahoma City Oklahoma USA; ^4^ Immunophotonics Inc. St. Louis Missouri USA; ^5^ Huntsman Cancer Institute University of Utah Salt Lake City Utah USA

**Keywords:** antitumour immune response, localized ablative immunotherapy (LAIT), mouse mammary tumour virus‐polyoma middle T (MMTV‐PyMT), N‐dihydrogalactochitosan (GC), photothermal therapy (PTT), single‐cell RNA sequencing (scRNAseq), tumour‐infiltrating T cells

## Abstract

**Background:**

Metastatic breast cancer poses great challenge in cancer treatment. N‐dihydrogalactochitosan (GC) is a novel immunoadjuvant that stimulates systemic immune responses when administered intratumourally following local tumour ablation. A combination of photothermal therapy (PTT) and GC, referred to as localized ablative immunotherapy (LAIT), extended animal survival and generates an activated B cell phenotype in MMTV‐PyMT mouse mammary tumour microenvironment (TME). However, how T cell populations respond to LAIT remains to be elucidated.

**Methods:**

Using depletion antibodies, we studied the contributions of CD8^+^ and CD4^+^ T cells to the therapeutic effect of LAIT. Using single‐cell RNA‐sequencing (scRNAseq), we analysed tumour‐infiltrating T cell heterogeneity and dissected their transcriptomes upon treatments of PTT, GC, and LAIT (PTT+GC).

**Results:**

Loss of CD8^+^ T cells after LAIT abrogated the therapeutic benefits of LAIT. Ten days after treatment, proportions of CD8^+^ and CD4^+^ T cells in untreated TME were 19.2% and 23.0%, respectively. Upon LAIT, both proportions were increased to 25.5% and 36.2%, respectively. In particular, LAIT increased the proportions of naïve and memory cells from a resting state to an activated state. LAIT consistently induced the expression of co‐stimulatory molecules, type I IFN responsive genes, and a series of antitumor cytokines, *Ifng*, *Tnf*, *Il1*, and *Il17* in CD8^+^ and CD4^+^ T cells. LAIT also induced immune checkpoints *Pdcd1, Ctla4*, and *Lag3* expression, consistent with T cell activation. Relevant to clinical translation, LAIT also upregulated genes in CD8^+^ and CD4^+^ T cells that positively correlated with extended survival of breast cancer patients.

**Conclusions:**

Overall, our results reveal that LAIT prompts immunological remodelling of T cells by inducing broad proinflammatory responses and inhibiting suppressive signalling to drive antitumour immunity.

## Graphical abstract

LAIT induced a broad proinflammatory response in tumour‐infiltrating T cells. LAIT causes tumour immunogenic cell death (ICD), releasing danger associated molecular patterns (DAMPs) and tumour antigens (TAs). DAMPs and TAs recruit T cells into the tumour microenvironment, and the GC‐induced cytokines drive their differentiation into T helper 1 and 17 (Th1 and Th17) cells and cytotoxic lymphocytes (CTL).

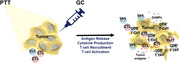

## 1 INTRODUCTION

Immune checkpoint inhibitors (ICI), which block the signalling of CTLA‐4 or PD‐1 on the surface of T cells or blocks the PD‐1 ligand (PD‐L1) on the surface of tumour cells, has been widely accepted in clinical applications.^1^, ^2^ However, its efficacy remains unsatisfactory with a ∼20% response rate across a wide range of cancers.^3^ There are several factors contributing to this failure, but a key component limiting the efficacy of cancer therapeutics is the tumour microenvironment (TME). The TME contains a diverse mixture of immune and non‐immune cells that can influence the efficacy of therapeutics either positively or negatively. Moreover several tumour intrinsic and extrinsic mechanisms play a substantial role in moderating tumour immune recruitment, stimulation, and activation.^4^ This is clearly illustrated in clinical responses to ICI in which a large percentage of the treated patients do not achieve long‐term tumour control.^5^, ^6^ Clinically, patients are grouped into three categories based on their responses to ICI therapies: primary resistance, adaptive resistance, and acquired resistance.^5^, ^7^, ^8^, ^9^ Primary resisters fail to respond to ICI, and it is hypothesized that either low tumour mutational burden or tumour peptide presentation leads to insufficient T cell responses, and/or an unfavourable TME actively prevents sufficient T cell mediated immune responses from killing the tumour. Adaptive immune resisters have sufficient antitumor T cell responses, but a hostile TME actively prevents sufficient tumour killing through a variety of tumour intrinsic and extrinsic mechanisms.^10^, ^11^ Last, there are also patients who initially respond to ICI but then relapse and the cancer progresses, resulting in acquired resistance. Hence, there is a great need to design a suitable therapeutic modality that not only recruits T cells into TME, but also enhances T cell activation while simultaneously disrupting the TME in such a way to promote antitumor immunity capable of synergizing with ICI and other immunotherapies.

A local intervention‐based approach that induces systemic, long‐term antitumor immunity represents an ideal therapy for metastatic cancers. Localized ablative immunotherapy (LAIT) was developed to achieve this objective. LAIT has two components: local photothermal therapy (PTT) and local administration of an immunostimulant, such as N‐dihydrogalactochitosan (GC).^12^, ^13^, ^14^, ^15^ Tumour ablation utilizes physical means, such as heat, to destroy a tumour locally and/or disrupt tumour homeostasis. Importantly, immunogenic cell death (ICD), resulting from ablation, releases tumour antigens, damage‐associated molecular patterns (DAMPs), as well as RNA and DNA, all of which stimulate dendritic cells to support inflammatory immune responses.^16^, ^17^, ^18^ Unfortunately, ablation alone is not enough to overcome the anti‐inflammatory TME and to promote sustainable antitumor immune responses. For this reason, we combined PTT with GC to enhance the immune stimulatory response established by PTT, promote antitumor inflammation, and generate long‐term antitumor immunity. In preliminary clinical studies for patients with metastatic, treatment‐recalcitrant breast cancer and melanoma, LAIT successfully reduced and/or eliminated the treated primary tumours and untreated metastases in the lungs.^19^, ^20^, ^21^ Due to the systemic antitumor responses observed, we hypothesize that LAIT activates sustainable adaptive antitumor immune responses. Supporting this notion, we previously reported that synergizing nanomaterial‐delivered PTT and GC with anti‐CTLA‐4 enhanced the LAIT cure rate and rendered the highly metastatic 4T1 breast tumuor model sensitive to ICI.^22^

To elucidate the factors contributing to T cell activation following LAIT, we used single‐cell RNA sequencing (scRNAseq) to evaluate the immunological effects of LAIT on breast tumours arising from the mouse mammary tumour virus‐polyoma middle T (MMTV‐PyMT) transgenic mice. MMTV‐PyMT tumours have high penetrance and emulate the histological stages of human luminal type B breast cancer.^23^, ^24^, ^25^ Moreover, this tumour model lacks expression of the oestrogen and progesterone receptor and overexpresses EbrB2 and cyclin D1 as the tumour progresses, which mimics human breast cancers with poor prognosis.^24^, ^26^ Immunologically, this tumour becomes infiltrated with a plethora of immune cells, predominantly macrophages and T cells, that contribute to metastases and progression,^27^, ^28^, ^29^ and it is largely unresponsive to ICI.^30^, ^31^, ^32^

In this study, we sought to determine if LAIT disruption of the established TME could alter the transcriptome of tumour‐infiltrating T cells and allow for immune mediated tumour killing. T cells from untreated (CTRL), PTT, GC, and LAIT (PTT+GC) treated MMTV‐PyMT tumours were evaluated. Single cell transcriptomes revealed a dynamic atlas of CD4^+^ and CD8^+^ T cells in the TME in response to treatment. Using single cell trajectory analysis, we uncovered that GC and LAIT treatment pushed the tumour resident T cells into an activated state from a resting state. Complementing these findings gene expression analysis also revealed that LAIT consistently induced genes that enriched pathways involved in co‐stimulation, responses to type I IFN, and antitumor cytokine production including *Ifng*, *Tnf*, *Il1*, and *Il17* across the T cell subtypes. In support of the activated T cell phenotype, we also observed induction of immune checkpoints of *Pdcd1* and *Ctla4*, indicating that LAIT has great potential to synergize with ICI. More clinically relevant, LAIT upregulated genes in CD8^+^ T and CD4^+^ T cells that positively correlated with extended survival of breast cancer patients. Overall, our results reveal that LAIT prompts immunological remodelling of T cells by inducing broad proinflammatory responses and inhibiting suppressive signalling to drive antitumor immunity. This study provides the rationale for synergizing LAIT with ICI to overcome the inhibitory mechanisms imposed by the TME, therefore increasing the therapeutic efficacy of both treatments.

## 2 RESULTS

### 2.1 LAIT enhances the activation and diversity of tumour‐infiltrating T cells

**FIGURE 1:**
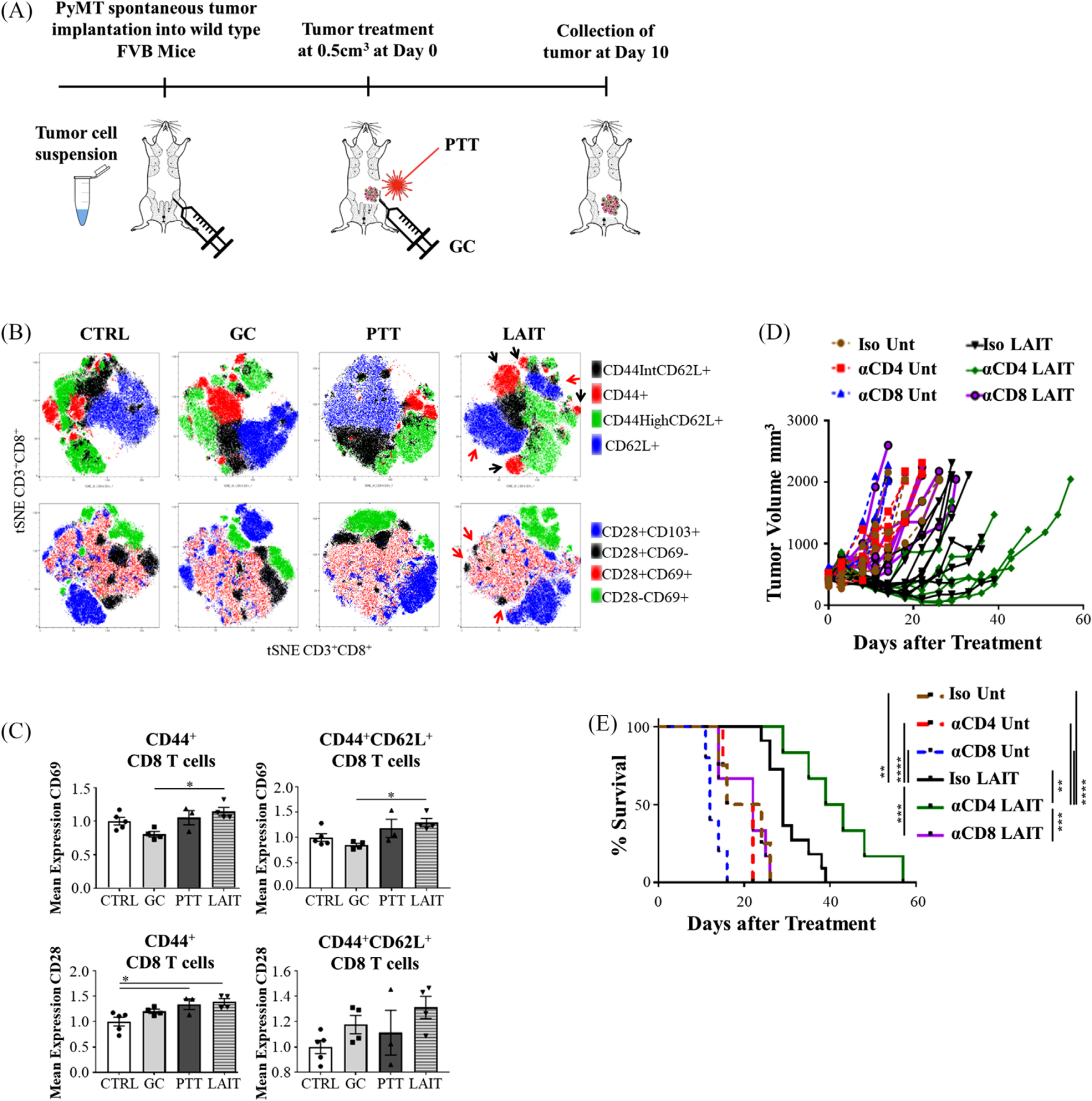
LAIT treatment slows tumour growth by activating tumour‐infiltrating CD8^+^ T cells. (A) Schematic of MMTV‐PyMT tumour implantation, treatment, and analysis. (B) T‐SNE plots generated from three individual concatenated files in FlowJo of tumour‐infiltrating CD8^+^ T cells from different treatment groups. Manual gates for CD44 and CD62L were overlayed onto the T‐SNE plots. The colours indicate the expression of different cell surface marker combinations. T cells were analysed 10 days after treatment. The black arrows in the upper panel highlight the CD44^+^CD8^+^ T cells. The red arrows in the upper panel highlight the CD62L^+^CD8^+^ T cells. In the lower panel, the red arrows are highlighting the CD28^+^CD69^–^CD8^+^ T cells. (C) The MFI of CD69 and CD28 on CD8^+^ T cells, normalised to the CTRL, 10 days after different treatments. One‐way ANOVA was used for statistical analysis. (D) Individual tumour size of untreated or LAIT‐treated mice that were injected with isotype antibodies or CD4^+^ or CD8^+^ depletion antibodies. (E) Survival rates of untreated or LAIT‐treated tumour‐bearing mice injected with isotype antibodies or depleted of CD4^+^ or CD8^+^ T cells. Log‐rank (Mantel‐Cox) test was used for statistical analysis.

Mice expressing the MMTV (mouse mammary tumour virus) promoter‐driven PyMT (polyoma virus middle T) tumour antigen develop breast cancer which closely resembles human pathogenesis.^33^ Here, we used the MMTV‐PyMT transgenic tumour model to investigate the effects of LAIT on T cells in the TME. Spontaneous MMTV‐PyMT tumour cells were isolated from MMTV‐PyMT transgenic mice as previously described^33^ and were injected into the mammary fat pad of wild type female FVB mice (Figure 1A). When tumours reached a size of 0.5 cm^3^, mice were divided into four treatment groups: Untreated controls (CTRL), PTT alone, GC alone, and LAIT (PTT+GC) (*n* = 3–4 per group). Since cytotoxic T cells are crucial to antitumor immune responses, we evaluated the extent of cytotoxic T cell induction by LAIT in the TME 10 days after treatment by flow cytometry. Thermal effects of laser irradiation reduce tumour size, resulting in significantly smaller tumours in the PTT and LAIT groups than that in CTRL and GC groups (Figure S1A). For comparison, tumour‐infiltrating leukocyte (TIL) cellularity was divided by tumour volume. The total normalized CD45^+^ cellularity/mm^3^ (Figure S1A), following the gating strategies shown in Figure S1B, showed no significant differences among different treatment groups. The effector cells (CD45^+^CD3^+^CD8^+^CD44^+^) and memory T cells (CD45^+^CD3^+^CD8^+^CD44^High^CD62L^+^) in LAIT and GC groups had a trending increase, while the CD45^+^CD3^+^CD8^+^CD44^Low^CD62L^+^ and CD45^+^CD3^+^CD8^+^CD62L^+^ naïve T cells in LAIT and GC groups were like that of CTRL and PTT groups (Figure S1A).

To further evaluate CD8^+^ T cell populations, using the gating strategy in Figure S1B, t‐distributed Stochastic Neighbor Embedding (t‐SNE) plots were generated from three concatenated samples from each group using FlowJo (Figure 1B and Figure S1C). These plots revealed more diversity within the CD8^+^ T cells from LAIT‐treated tumours based on their distribution and number of distinct clusters (Figure 1B). For example, the CD62L^+^ cells in the LAIT‐treated tumours separated into two distinct groups (blue clusters, red arrows). The effector cells (red clusters, black arrows) from the LAIT‐treated tumours also separated into distinct clusters, with a much greater distance between them on the t‐SNE plot (Figure 1B), again suggesting more T cell diversity. There was also a large reduction in the proportion of CD28^+^CD69^–^ cells (black clusters, red arrows) and an increase in the CD28^+^CD69^+^ cells (red dots) in the LAIT‐treated tumours, suggesting the differentiation and activation of CD8^+^ T cells following LAIT (Figure 1B). The intensity and distribution of CD69^+^, CD28^+^, and CD103^+^ expression on CD8^+^ T cells were all increased in the LAIT‐treated tumours (Figure S1C). The mean expression levels of CD69 (normalized to CTRL), as determined by mean fluorescence intensity (MFI), on the CD3^+^CD8^+^CD44^+^ and CD3^+^CD8^+^CD44^+^CD62L^+^ (including both CD44^High^CD62L^+^ and CD44^Low^CD62L^+^ populations) T cell subsets, were statistically elevated in the LAIT‐treated tumours compared to GC alone and trending up compared to CTRL and PTT (Figure 1C). CD28 was also significantly increased on the CD3^+^CD8^+^CD44^+^ T cells and trending up on the CD3^+^CD8^+^CD44^+^CD62L^+^ T cell subsets (Figure 1C). These results indicate that the CD8^+^ T cells are activated in response to LAIT, but this analysis did not fully explain the unique effects of LAIT on tumour growth inhibition.^34^

We next investigated the contributions of CD8^+^ and/or CD4^+^ T cells to the therapeutic effect of LAIT, using depletion antibodies (Figure S1D), with >95% depletion confirmed by flow cytometry (data not shown). Loss of CD8^+^ T cells after LAIT treatment abrogated the therapeutic benefits of LAIT, suggesting that cytotoxic T lymphocytes (CTLs) are critical for downstream responses following LAIT (Figure 1D,E). Loss of CD4^+^ T cells following LAIT treatment had the opposite effect, resulting in delayed tumour growth and prolonged survival in a manner superior to LAIT alone (Figure 1E). This is not unexpected as CD4^+^ T regulatory (Tregs) cells play a significant role in tumour progression and metastases, as loss of CD4^+^ T cells prohibited lung metastases in untreated MMTV‐PyMT tumour bearing animals.^35^ These findings also support the notion that CD8^+^ T cells are imperative to mounting an anti‐tumour response in this model. This should not diminish the important role of CD4^+^ T cells in generating T cell immunity but indicates that the timing of CD4^+^ T cell depletion likely changed the dependence on CD4^+^ T cells in this model. Moreover, this is a virally driven oncogene model, and as such a strong CD8^+^ T cell response likely exists well before the depletion of CD4^+^ T cells as these tumours were implanted first and allowed to reach a specific size before T cell depletion. Thus, the help of CD4^+^ T cells is not as critical for acute stimulation of a memory response, as corroborated by individual tumour growth in which the CD4^+^ T cell depleted animals had significant tumour regression initially (Figure 1D). However, it has been well documented that CD4^+^ T cells are required to maintain CD8^+^ T cell function during chronic infections and/or chronic immune stimulation.^36^, ^37^ This is also reflected in the tumour regrowth in the CD4^+^ depleted animals (Figure 1D). Whether the CD8+ T cells become anergic or die off in the absence of CD4^+^ T cells is the subject of future work.

### 2.2 ScRNAseq reveals diversity of tumour‐infiltrating T cells upon LAIT

**FIGURE 2:**
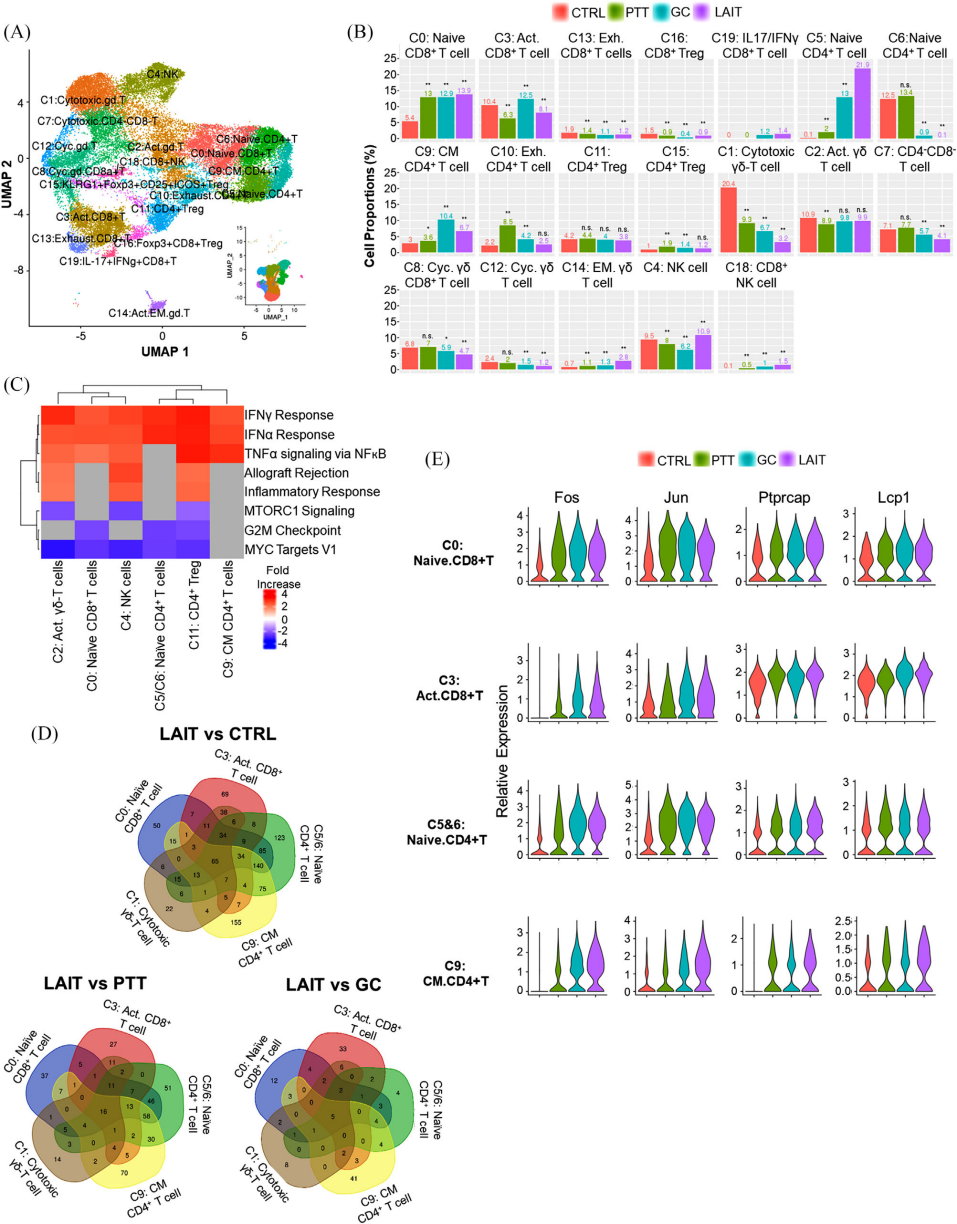
ScRNAseq analysis of CTRL, PTT, GC, and LAIT treated tumours creates a tumour‐infiltrating immune cell atlas. (A) UMAP of re‐clustered lymphoid cells with an SNN resolution of 0.7. (B) Proportions of re‐clustered lymphoid cells in different treatment groups. (C) Heatmap of pathway enrichment for lymphoid cell clusters induced by LAIT using GSEA. (D) Venn diagrams showing the number of DEGs and commonly expressed genes in clusters 0, 3, 5/6, 1, and 9. (E) Expression levels of four representative DEGs identified in the Venn diagrams from clusters 0, 3, 5/6, and 9.

To investigate a broad spectrum of immune cell compositions and transcriptional profiling in the TME 10 days after treatment, scRNAseq analyses were performed following the workflow depicted in Figure S2A. TILs were collected and enriched for CD45^+^ cells (4 mice for each group). CD45^+^ cells from two separate animals were pooled together in equal numbers and then used for sequencing. First, all 49 380 TILs (11 584 CTRL; 14 071 PTT; 12 501 GC; 11 224 LAIT) were integrated for scRNAseq by *Seurat* R package.^38^ To focus on the effects of LAIT on T cells, we extracted the lymphoid cell populations (T cells plus NK cells), performed quality control and cluster filtering, and obtained 17 T‐cell and 2 NK‐cell subpopulations/clusters after re‐clustering analysis using the default shared‐nearest neighbour (SNN) as shown by Uniform Manifold Approximation and Projection (UMAP) in Figure 2A. The unsupervised analysis of the top 10 differentially expressed genes (DEGs) from each cell confirmed that the tumour‐infiltrating lymphoid compartment cells were successfully delineated into 19 distinct clusters as visualized by the heatmap (Figure S2B).

LAIT significantly upregulated naïve CD8^+^ T cells (cluster 0) and central‐memory CD4^+^ T cells (cluster 9), as shown in Figure 2B. Clusters 5 and 6 both contained naïve CD4^+^ T cells (Figure 2B). Additionally, LAIT decreased the cell ratio of cluster 1 (cytotoxic γδ‐T cells) and induced a small proportion of IL‐17^+^IFNγ^+^ CD8^+^ T cells in cluster 19 (Figure 2B). The appearance of naïve T cells leads us to hypothesize that LAIT can potentially recruit these cells to the TME and potentially generate *de novo* T cell responses, not just enhance existing T cells responses. This is significant as LAIT has the potential to generate strong, more diverse T cell pool that limits exhaustion and immune evasion.

To determine how the lymphoid cells are activated, we performed gene set enrichment analysis (GSEA). Using differentially expressed genes (DEGs), we focused on the comparison between the LAIT and CTRL groups. As shown in Figure 2C, LAIT significantly upregulated IFNα and IFNγ signalling pathways of activated γδ‐T cells (cluster 2), naïve CD8^+^ T cells (cluster 0), NK cells (cluster 4), the combined naïve CD4^+^ T cells (cluster 5 and 6), CD4^+^ Tregs (cluster 11), and central memory CD4^+^ T cells (cluster 9). Furthermore, mTORC1, G2M checkpoint, and Myc target pathways were downregulated by LAIT in these cell clusters (Figure 2C). The signalling pathway analysis demonstrates that LAIT promotes the antitumor type I/II IFNs and TNF pathways across different lymphoid cell types.

To find the common DEGs in five separate lymphoid cell clusters from different comparison groups, we generated Venn diagrams (Figure 2D). We compared CD8^+^ T cells from clusters 0 and 3 with CD4^+^ T cells from clusters 5, 6, and 9, and γδ T cells in cluster 1. In comparison, 65, 16, and 5 commonly shared DEGs were found in LAIT versus CTRL, LAIT versus PTT, and LAIT versus GC, respectively (Figure 2D; Table S1). We also generated violin plots for a series of conserved DEGs for LAIT versus CTRL in these selected T cell types and found that LAIT significantly promoted the expression of several genes involved in T cell activation, function, and/or survival (Figure 2E; Figure S2D and Table S1). For example, LAIT promoted the expression of Gimap transcripts, *Dusp1 (MKP‐1), Isg15*, and *Ptprc*, which are involved in T cell survival, activation, and death (Figure S2D).^39^, ^40^, ^41^, ^42^ LAIT also induced the expression of *Fos, Jun, Ptprcap*, and *Lcp1* (Figure 2E), which are associated with T cell receptor (TCR) signaling,^43^, ^44^, ^45^ indicating that T cells in LAIT‐treated tumours are responding to their cognate antigens. LAIT also initiated changes in several other genes with less known functions in T cells, as listed in Table S1.

### 2.3 LAIT promotes naïve and memory T cell activation in the TME

**FIGURE 3:**
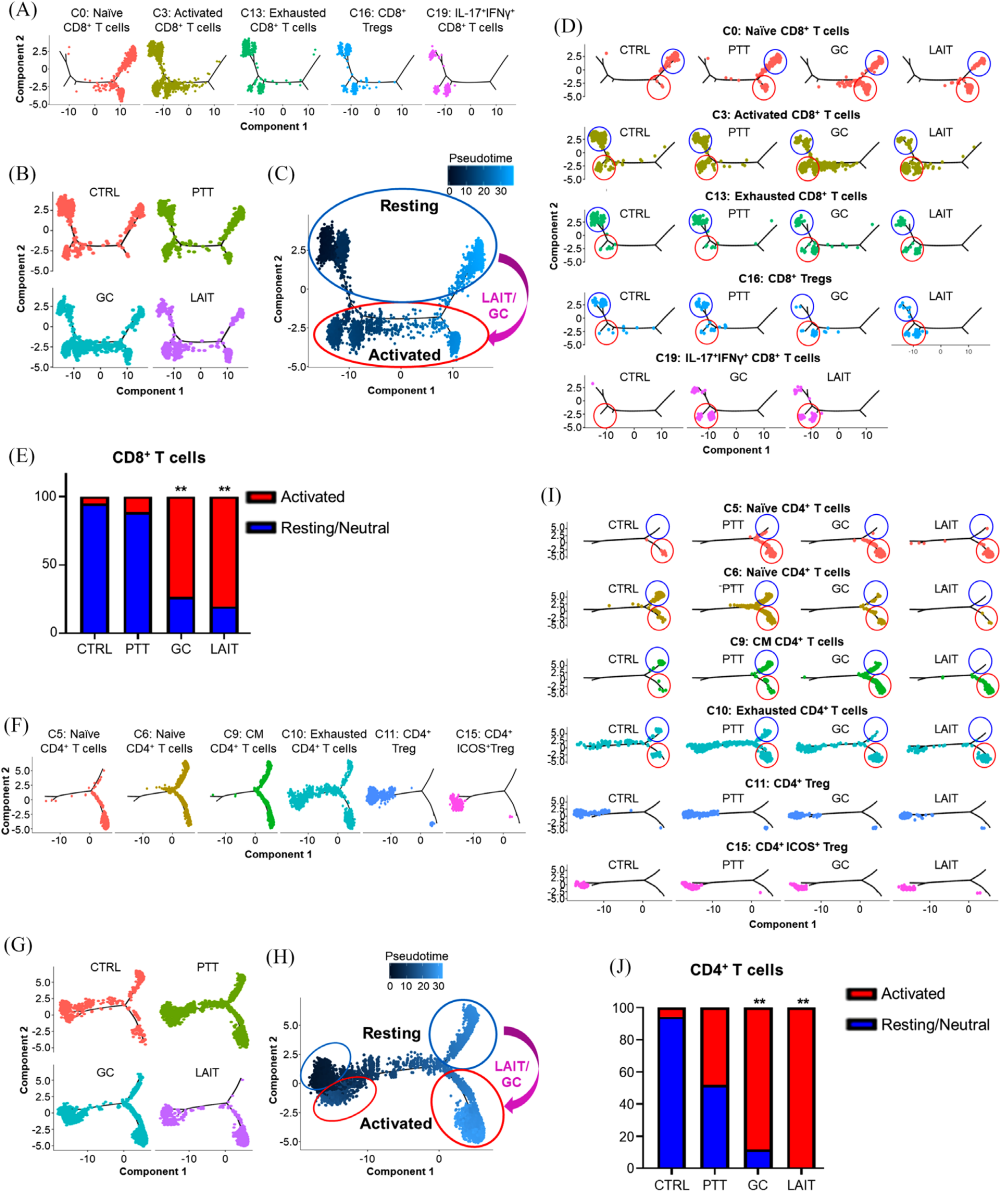
T cells from LAIT‐treated tumours reside in activated states. (A) Branched trajectory of CD8^+^ T cells in different clusters separated according to each annotated cell type. (B) Branched trajectory of CD8^+^ T cells separated according to treatment group. (C) LAIT/GC induced transitioning (purple arrow) pattern for CD8^+^ T cells from a resting state (blue circle) to an activated state (red circle). (D) Branched trajectories of CD8^+^ T cell subtypes in different treatment groups. Cells in blue circles are in a resting state and cells in red circles are in an activated state. (E) CD8^+^ T cell proportions from resting and activated trajectories in different treatment groups. Statistical analysis was performed using proportion test function (prop.test) in R. **p < .01. (F) Branched trajectory of CD4^+^ T cells in different clusters separated according to each annotated cell type. (G) Branched trajectory of CD4^+^ T cells separated according to treatment group. (H) LAIT/GC induced transitioning (purple arrow) pattern for CD4^+^ T cells from a resting state (blue circle) to an activated state (red circle). (I) Branched trajectories of CD4^+^ T cell subtypes in different treatment groups. Cells blue circle are in a resting state and cells in red circles are in an activated state. (J) CD4^+^ T cell proportions from resting and activated trajectories in different treatment groups. Statistical analysis was performed using proportion test function (prop.test) in R. **p < .01.

To confirm that T cells in LAIT‐treated tumours were activated, we performed cell trajectory inference (CTI) analysis, using R package *Monocle2*,^46^ on CD8^+^ (Figure 3A–D and Figure S3A) and CD4^+^ T cells (Figure 3F–I and Figure S3C). The CD8^+^ T cells were divided into 9 states (Figure S3A) with the naïve CD8^+^ T cells distributed on the right branches, including states 4, 5, and 6 (Figure 3A and Figure S3A). Non‐naïve subtypes of CD8^+^ T cells were distributed on the left branches, including states 1, 2, 3, 7, 8, and 9 (Figure 3A and Figure S3A). When separated based on different treatments, the cell trajectory patterns were shifted by GC and LAIT from upper states (1, 2, 3, 5, 9) to lower states (6, 7, 8), as shown in Figure 3B. To determine if the shift was indeed due to T cell activation, we generated volcano plots of the top DEGs of CD8^+^ T cells between the LAIT‐treated and CTRL tumours (Figure S3B). The genes highly enriched in cluster 0, 3, and 13 CD8^+^ T cells from LAIT‐treated tumours were associated with T cell activation such as *Fos, Jun, Pcdc1, Egr1, and Dusp1* or type I IFN responses such as *Ly6a*, and *Ifi27l2a* (Figure S3B). These genes complement the activation and TCR signalling genes highlighted in Figure 2 and Figure S2. Thus, we conclude that cells in the upper branches are in a resting/neutral state (blue circle) and the cells in lower branches are in an activated state (red circles), as shown in Figure 3C,D. By examining individual CD8^+^ T cell clusters, we observed a shift in the CD8^+^ T cells in clusters 0, 3, 13, and 16 towards the activated states in GC and LAIT‐treated tumours (Figure 3D). Overall, the proportion of CD8^+^ T cells in the activated state increased dramatically in the LAIT‐treated tumours (Figure 3E).

We also performed CTI analysis on CD4^+^ T cells (Figure 3F and Figure S3C). CD4^+^ T cells were divided into 11 states (Figure S3C). Naïve and central memory CD4^+^ T cells were distributed on the right branches, which included states 6 through 11 (Figure 3F and Figure S3C). Exhausted CD4^+^ T cells showed ubiquitous expression throughout the trajectory. The Tregs were localized at the left branches, in an opposite pattern to that of naïve CD4^+^ T cells (Figure 3F). When separated based on treatment groups, the cell trajectory patterns were shifted by GC and LAIT from the upper states to lower states (Figure 3G), like that of CD8^+^ T cells (Figure 3B). To confirm this switch was also due to T cell activation, volcano plots from DEGs of CD4^+^ T cells between LAIT‐treated and CTRL tumours were generated (Figure S3D). The genes associated with T cell activation were upregulated in LAIT‐treated tumours similarly to the CD8^+^ T cells (Figure S3D). Based on the gene expression data, we labelled the cells residing in the lower states (red circle) as activated and cells residing in the upper states (blue circles) as resting/neutral (Figure 3G–I).

GC and LAIT shifted CD4^+^ T cells towards the activated state in clusters 5, 6, 9, and 10 (Figure 3I). Using our clustering analysis of the lymphoid cells, we noted that even though clusters 5 and 6 contained both naïve CD4^+^ T cells based on their expression of *Sell* and *Il7r* (Figure S2B), the cells in cluster 5 were activated while cells in cluster 6 were resting/neutral. This likely explains the dramatic shift in these two populations by LAIT (Figure 2B) as nearly all the T cells in cluster 5 reside in the activated state (red circles) along with very few T cells from cluster 6 in the LAIT‐treated tumours (Figure 3I). Interestingly, only in the LAIT‐treated tumours we observed that all the central memory and exhausted T cells (cluster 9 and 10) resided in the activated state (Figure 3I). The exhausted cell phenotype was still characterized as such due to the expression of *Tox*, *Ctla4*, and *Slamf6*, but the fact that these cells can shift from resting to activated suggests that the exhausted phenotype is not permanent in these cells. Moreover, the proportions of total CD4^+^ T cells residing in the resting/neutral state versus the activated state was significantly different in the GC and LAIT‐treated tumours compared to that in the CTRL and PTT‐treated tumours (Figure 3J).

To determine how LAIT is globally affecting CD8^+^ and CD4^+^ T cell activation, we performed GSEA on total CD8^+^ or CD4^+^ T cells from the LAIT‐treated and CTRL tumours. LAIT significantly upregulated proinflammatory signalling pathways IFNα, IFNγ, and TNFα and downregulated Myc targets and G2M checkpoint regulators in both CD8^+^ (Figure S3E) and CD4^+^ (Figure S3F) T cells.

### 2.4 LAIT selectively induces type I IFN transcriptional responses in CD8^+^ T cells

**FIGURE 4:**
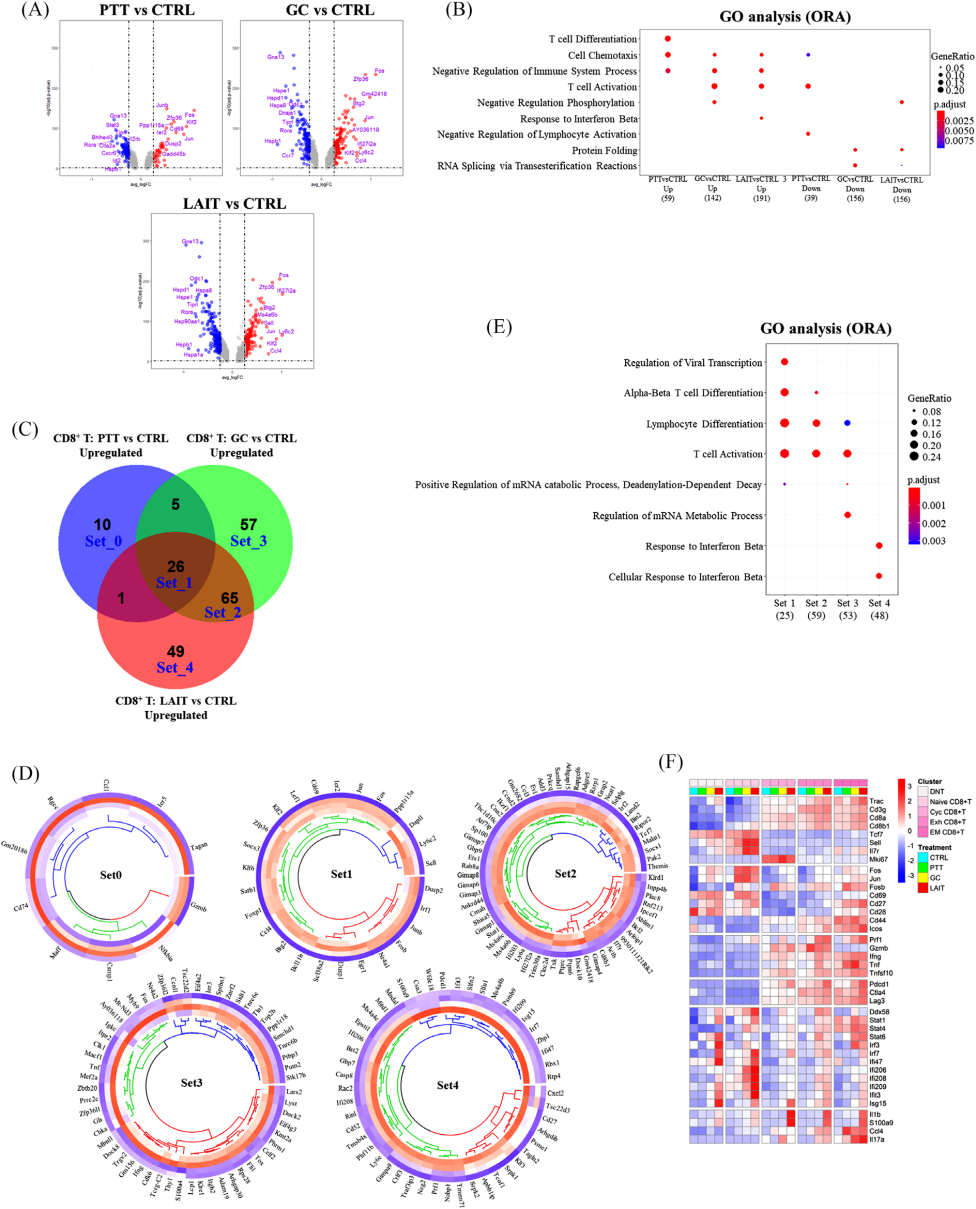
Analyses of differential gene expression, pathway enrichment, gene set overlapping for tumour‐infiltrating CD8+ T cell populations. (A) Volcano plot showing differential gene expression comparing different treatment groups: PTT versus CTRL, GC versus CTRL, and LAIT versus CTRL in CD8^+^ T cells. Top 10 upregulated (red) and downregulated (blue) genes are labelled. (B) Dot plot showing biological process (BP) of gene ontology (GO) analysis using over‐representation analysis (ORA) method for both upregulated (up) and downregulated (down) genes for PTT versus CTRL, GC versus CTRL, and LAIT versus CTRL. (C) Venn diagram showing upregulated genes from comparisons of PTT versus CTRL, GC versus CTRL, and LAIT versus CTRL. Five gene sets with large number of overlapping genes, from Set_0 to Set_4, are labelled. (D) Circular heatmap showing the expression of genes from upregulated Set_0 to Set_4 in (C). Heatmap columns for groups of CTRL, PTT, GC, and LAIT were arranged from outside to inside. Higher expression was coloured in red while lower in blue. (E) Dot plot for BP of GO analysis of upregulated genes in Set_1 to Set_4. (F) Heatmap showing the expression of selected genes in each treatment group in each CD8^+^ T cell subtype.

To highlight the transcriptional changes that occur between each treatment group and to understand why LAIT, but not GC alone, is able to control tumour growth, we next explored differential gene expression and functional enrichment analysis on the tumour‐infiltrating CD8^+^ T cells. Three comparisons were made: PTT versus CTRL, GC versus CTRL, and LAIT versus CTRL. For each comparison, differentially expressed genes (DEGs) were generated by using *FindMarkers* function in *Seurat* R package.^38^ DEGs from comparing two treatment groups were defined as log Fold change >0.25 or < –0.25 along with adjusted *p* value <.05, shown as red and blue dots in the volcano plots (Figure 4A). Functional enrichment analyses were performed using Gene Ontology (GO)^47^ based on the over‐representation method (ORA)^48^ using *clusterProfiler* R package.^49^

For PTT versus CTRL, 42 upregulated and 75 downregulated genes were shown in the volcano plot (Figure 4A). For pathway enrichment analysis, 40 upregulated and 67 downregulated genes were used after filtering (Figure 4B). Biological process (BP) of gene ontology (GO) analysis using the ORA method demonstrated that PTT induced T cell differentiation and cell chemotaxis pathways compared to CTRL (Figure 4B). GC stimulation upregulated 153 genes and downregulated 137 genes compared to CTRL (Figure 4A). GO analysis revealed that GC upregulated genes were enriched in T cell activation and negative regulation of phosphorylation. When comparing LAIT versus CTRL, we observed 141 upregulated and 204 downregulated genes (Figure 4A). LAIT‐induced genes were enriched in T cell activation and response to IFN‐β (Figure 4B), suggesting an anti‐tumour function. Both GC and LAIT downregulated genes involved in protein folding and RNA splicing processes (Figure 4B).

We hypothesized that understanding the relationships among the treatment regulated DEGs would shed light on the molecular mechanism of LAIT. To address this, we used the Venn diagram to display the overlap of PTT, GC, and LAIT regulated genes (Figure 4C). Genes regulated by PTT alone were confined in Set_0. PTT‐GC‐LAIT intersecting genes were confined in Set_1; GC‐LAIT intersecting genes (excluding Set_1) were confined in Set_2. Set_0, Set_3, and Set_4 were defined as specific upregulation by PTT, GC, and LAIT, respectively. Collectively, PTT, GC, and LAIT specifically upregulated 10 (Set_0), 57 (Set_3), and 49 (Set_4) genes (Figure 4C). Here, we defined Set_1 as LAIT's additive effect gene sets because expression of genes in this set were all significantly increased by PTT, GC, and LAIT when compared with CTRL (Figure 4D). For Set_2, gene expression levels in GC and LAIT groups (the inner circles in Figure 4D) were higher than PTT or CTRL groups (the inner circles in Figure 4D). Set_4 was defined as LAIT's synergistic effect gene set since statistically significant gene expression was only found in LAIT group (the innermost circles in Figure 4D), but not in PTT or GC alone groups, when compared to CTRL.

GO analysis for the individual gene sets were shown in Figure 4E. Biological processes (BP), such as alpha‐beta T cell differentiation and T cell activation were enriched in gene Set_1, 2, and 3, which is common to all three treatments (Figure 4E). LAIT‐specific (Set_4) pathways were enriched for response to IFNβ (Figure 4E).

To further investigate the transcriptional regulations of LAIT on finely identified cell subpopulations, we selected a panel of representative genes from the upregulated gene sets (Figure 4B) and compared their expressions in each treatment group across CD8^+^ T subtypes (Figure 4F). Effector marker *Cd44* and co‐stimulatory molecule *Icos* were expressed in non‐naïve clusters, with only *Icos* being enriched by LAIT in effector memory CD8^+^ T cell subtype. Cytotoxic molecule *Prf1* was strongly induced by LAIT in exhausted and effector memory CD8^+^ T cells, while *Gzmb* was only strongly promoted by PTT in exhausted T cell. *Ifng*, *Tnf* and *Tnfsf10* (TRAIL) were enriched in exhausted, cycling exhausted, and effector memory CD8^+^T clusters, with LAIT only slightly elevating levels of *Ifng* and *Tnfsf10*. Co‐inhibitor molecules of *Pdcd1* (PD1), *Ctla4* (CTLA4), and *Lag3* (LAG3) were mainly expressed in exhausted, cycling exhausted and effector memory CD8^+^ T clusters with *Pdcd1* showing slight induction by LAIT (Figure 4F). This is consistent with an exhausted and activated phenotype. More importantly, this data provide a rationale for combining defined ICI with LAIT to enhance T cell activation, with the hope of sustaining T cell mediated tumour killing to completely eliminate the tumours and prevent relapse.

Anti‐viral pathway component *Ddx58* (RIG‐I) was ubiquitously expressed and sharply upregulated by LAIT in naïve and memory CD8^+^ T cells. Downstream molecules, including transcription factors *Stat1*, *Stat4*, and *Stat6*, were activated by treatments of PTT, GC, and LAIT with variable degrees. *Irf3* and *Irf7* were strongly induced by LAIT in activated and naïve memory CD8^+^ T cells, respectively. Interferon induced genes, including *Ifi47*, *Ifi206*, *Ifi208*, *Ifi209*, and *Ifit3*, were markedly stimulated by LAIT in naïve memory CD8^+^ T cells. Interferon stimulated genes *Isg15* showed high promotion by LAIT in naïve memory, activated and cycling CD8^+^ T cells. In exhausted and effector memory T cells, *Ccl4* and *Il17a* were upregulated by treatments of PTT, GC, and LAIT, and proinflammatory genes *Il1b* and *S100a9* were also strongly induced by LAIT. These results illustrate that LAIT selectively stimulates T cell activation and IFN related pathway genes in CD8^+^ T cell subpopulations (Figure 4F).

We also investigated the relations of downregulated genes by PTT, GC, LAIT (Figure S4A), displayed the expression of gene sets (Figure S4B), and explored their gene ontology enrichment (Figure S4C). Treatment‐downregulated genes were mainly involved in the regulation of lymphocyte proliferation, protein folding, and receptor signalling pathway (Figure S4C). Similarly, we detected the selected downregulated genes in each treatment in each CD8^+^ T cell subtype (Figure S4D). Specifically, LAIT downregulated expressions of immune suppressing molecule *Tgfb1* and related genes, including *Lrrc32*, *Foxo1*, *Foxo3*, *Stat5a*, *Id2* and *Hif1a*. *Hspa1a* and *Hspb1* were also downregulated by LAIT across the CD8^+^ T subpopulations (Figure S4D).

### 2.5 LAIT selectively induces type I/II IFN transcriptional profiles in CD4^+^ T cells

**FIGURE 5:**
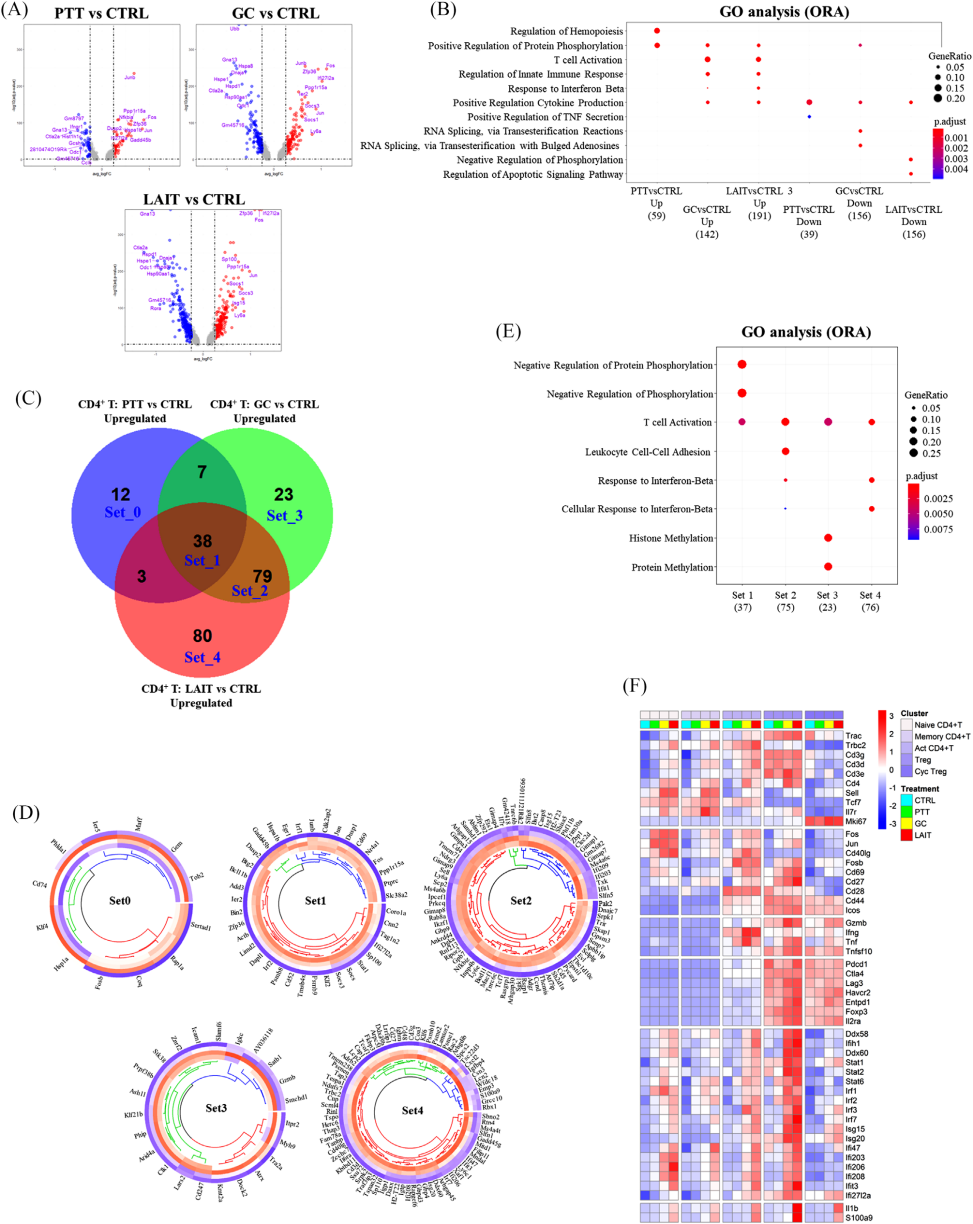
Analyses of differential gene expression, pathway enrichment, and gene set overlapping for tumour‐infiltrating CD4^+^ T cell populations. (A) Volcano plot showing differential gene expression comparing different treatment groups: PTT versus CTRL, GC versus CTRL, and LAIT versus CTRL in CD4^+^ T cells. Top 10 upregulated (red) and downregulated (blue) genes are labelled. (B) Dot plot showing biological process (BP) of gene ontology (GO) analysis using over‐representation analysis (ORA) method for both upregulated (up) and downregulated (down) genes for PTT versus CTRL, GC versus CTRL, and LAIT versus CTRL. (C) Venn diagram showing upregulated genes from comparisons of PTT versus CTRL, GC versus CTRL, and LAIT versus CTRL. Five gene sets with large number of overlapping genes, from Set_0 to Set_4, are labelled. (D) Circular heatmap showing the expression of genes from upregulated Set_0 to Set_4 in (C). Heatmap columns for groups of CTRL, PTT, GC, and LAIT were arranged from outside to inside. Higher expression was coloured in red while lower in blue. (E) Dot plot for BP of GO analysis of upregulated genes in Set_1 to Set_4. (F) Heatmap showing the expression of selected genes in each treatment group in each CD4^+^ T cell subtype.

To investigate the transcriptional changes induced by LAIT in helper T cells, the DEGs from PTT, GC, and LAIT treatment groups were produced for tumour‐infiltrating CD4^+^ T cells (Figure 5A). GC and LAIT induced more upregulated and downregulated genes than PTT (Figure 5B). Functional enrichment analysis showed that LAIT upregulated genes were involved in pathways relating to T cell activation and IFNβ responses, and downregulated genes involved in regulation of phosphorylation and apoptosis (Figure 5B).

To dissect the similarity and differences from treatments of PTT, GC, and LAIT on transcriptomic regulations in CD4^+^ T cells, we compared the upregulated and downregulated DEGs, respectively (Figure 5C and Figure S5A). PTT, GC, and LAIT shared a set of genes (Set_1) which were enriched in T cell activation pathways (Figure 5D). Genes from Set_4 had the highest expression in LAIT, supporting the synergistic effect between PTT and GC (Figure 5D). Moreover, genes specifically upregulated by LAIT were enriched in pathways of T cell activation and responses to IFNβ (Figure 5E).

Next, we sought to investigate the transcriptional changes of representative genes in each CD4^+^ T cell cluster responding to LAIT (Figure 5F). The cytotoxic molecule *Gzmb* was increased by GC in Tregs and cycling Tregs. *Ifng*, *Tnf* and *Tnfsf10* (TRAIL) were enriched in non‐naïve CD4^+^ T cell types, with *Ifng* being strongly driven by GC and LAIT. In activated CD4^+^ T cells, *Ifng* and *Tnf* was strongly induced by GC and LAIT suggesting that these treatments drive a T helper 1 response in activated T cells (Figure 5F). Co‐inhibitor molecules of *Pdcd1* (PD1), *Ctla4* (CTLA4), *Lag3* (LAG3), *Havcr2* (TIM3), and *Entpd1* (CD39) were mainly expressed in non‐cycling and cycling Treg clusters with *Lag3*, *Havcr2*, and *Entpd1*, showing slight induction by GC and LAIT. Treg genes *Foxp3* and *Il2ra* (CD25) also showed a similar regulation pattern. Type I IFN response in Tregs has been shown to mitigate Treg functionality.^50^, ^51^ However, our scRNAseq data also revealed elevated levels of *Gzmb*, cytotoxic cytokines, and ICIs suggesting that the tumour Tregs were also highly activated by our treatment and thus work against immune mediated killing of the tumour cells. This hypothesis was corroborated by the fact that CD4 depletion prior to LAIT treatment enhances tumour killing for a period (Figure 1D). These findings support future work for combining Treg inhibitors with LAIT to enhance tumour killing.

Anti‐viral pathway component *Ddx58* (RIG‐I) and *Ifih1* (MDA5) were ubiquitously expressed and stimulated by all treatments with sharp upregulation by LAIT in all subtypes of CD4^+^ T cells. Their positive regulator *Ddx60*, downstream molecules, including transcription factors of *Stat1*, *Stat2*, *Stat6*, *Irf1*, *Irf2*, *Irf3* and *Irf7*, were activated by treatments of PTT, GC, and LAIT with variable degrees. Interferon stimulated and induced genes, including *Isg15*, *Isg20*, *Ifi47*, *Ifi203*, *Ifi206*, *Ifi208*, *Ifit3*, and *Ifi27l2a*, showed similar regulation pattern by treatments and were markedly stimulated by GC and LAIT in all subpopulations of CD4^+^ T cells (Figure 5F).

In parallel, we investigated the relations of downregulated genes in CD4^+^ T cells by PTT, GC, and LAIT (Figure S5A), displayed the expression of gene sets (Figure S5B,C), and explored their gene ontology enrichment (Figure S5C). LAIT‐downregulated genes were enriched for regulation of protein phosphorylation (Figure S5C). Similarly, we detected the selected downregulated genes of each treatment in the CD4^+^ T cell subtypes (Figure S5D). LAIT downregulated expressions of a series of suppressing molecules, including *Lrrc32*, *Adora2a*, *Tgfb1*, *Nt5e* (CD73), in activated CD4^+^ T cells, Tregs and cycling Tregs. *Tgfbr2* was downregulated by LAIT in all subtypes of CD4^+^ T cells. Inhibition of *Foxo1*, *Foxo3*, *Hif1a*, *Stat3*, *Stat5a* by treatments of PTT, GC, or LAIT were seen in almost all subsets of CD4^+^ T cells, except that *Stat5a* showed a slight increase in cycling Tregs. Another panel of heat shock proteins, including *Hspa1a*, *Hspe1*, *Hspa8*, *Hspa4*, *Hspd1*, and *Dnajb6*, were also downregulated by treatments, especially by LAIT, across all the CD4^+^ T subpopulations (Figure S5D).

### 2.6 LAIT‐upregulated genes in CD8^+^ and CD4^+^ T cells positively correlate with greater overall survival of breast cancer patients

**FIGURE 6:**
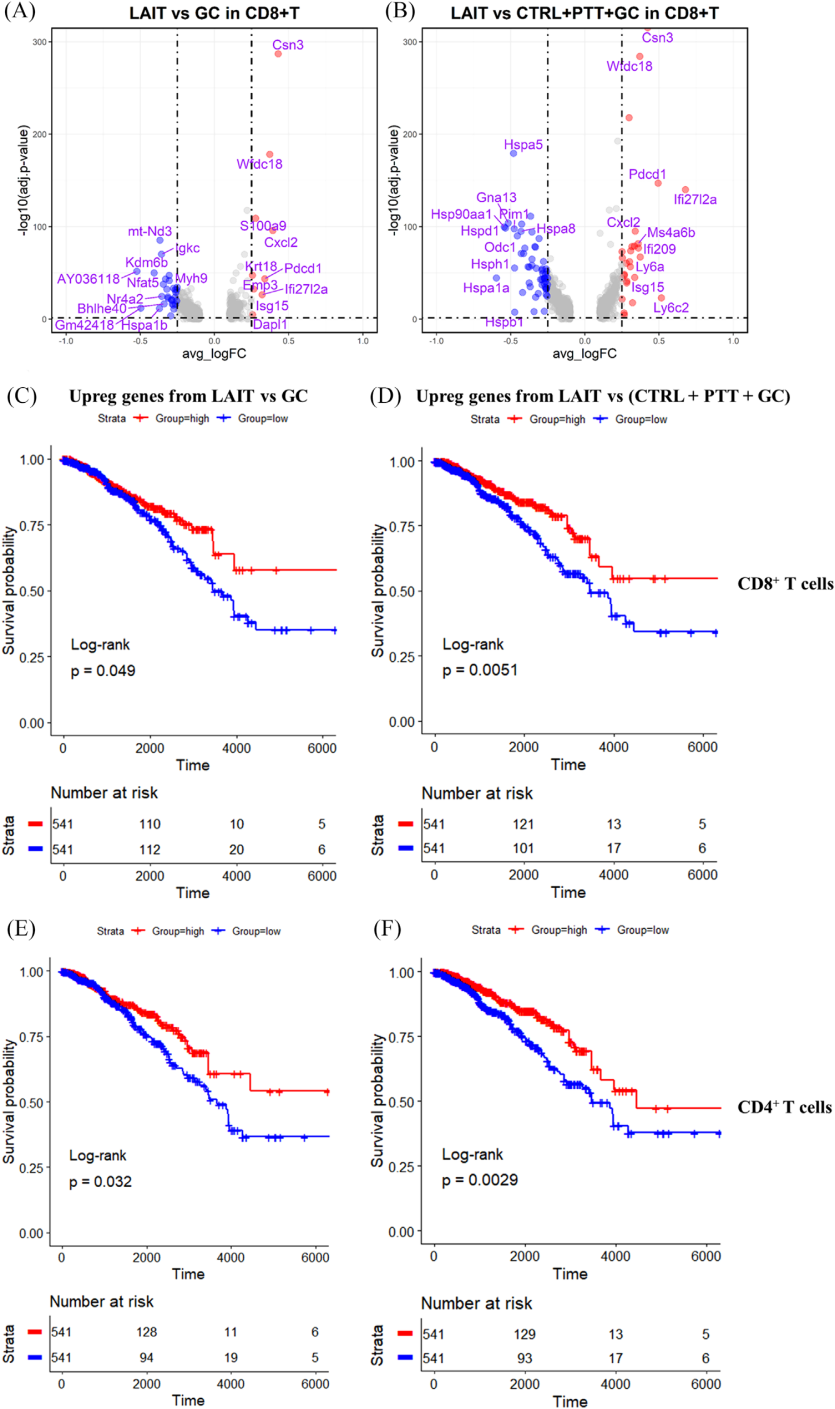
Association of LAIT specifically upregulated genes in T cell subtypes with breast cancer patient survival. (A) Volcano plot showing differential gene expression comparing LAIT with GC in CD8^+^ T cells. Top 10 upregulated (red) and downregulated (blue) genes are labelled. (B) Volcano plot showing differential gene expression comparing LAIT with other 3 groups (CTRL+PTT+GC) in CD8^+^ T cells. Top 10 upregulated (red) and downregulated (blue) genes are labelled. (C) Kaplan–Meier plots showing the significant difference in survival time (days) between breast cancer patients in groups with “high” and “low” expressions of LAIT versus GC‐derived upregulated genes from CD8^+^ T cells. (D) Kaplan–Meier plots showing the significant difference in survival time (days) between breast cancer patients in groups with “high” and “low” expressions of LAIT versus other 3 groups (LAIT vs CTRL+PTT+GC)‐derived upregulated genes from CD8^+^ T cells. (E) Kaplan–Meier plots showing the significant difference in survival time (days) between breast cancer patients in groups with “high” and “low” expressions of LAIT versus GC‐derived upregulated genes from CD4^+^ T cells. (F) Kaplan–Meier plots showing the significant difference in survival time (days) between breast cancer patients in groups with “high” and “low” expressions of LAIT versus other 3 groups (LAIT vs CTRL+PTT+GC)‐derived upregulated genes from CD4^+^ T cells

The above results showed that GC drove IFN response pathway gene signatures. The combination of PTT with GC augmented the antitumor IFN responses more than GC alone and achieved better therapeutic efficacy. To dissect the clinical relevance of the specific and synergistic effect of PTT and GC, we obtained both upregulated and downregulated gene signatures in CD8^+^ and CD4^+^ T cells by comparing LAIT to GC and comparing LAIT to combined CTRL, PTT, and GC groups (Figure 6A,B; Tables S2 and S3 and Figure S6A,B), respectively. For example, we examined whether the efficacy of LAIT in mouse breast tumours could translate to human disease by determining the relationship between the expression level of the LAIT versus GC upregulated genes and clinical outcomes in breast cancer patients. We utilized these upregulated and downregulated genes in mouse CD8^+^ and CD4^+^ T cells, mapped them into human counterparts, and obtained the enrichment score (ES) based on gene set variation analysis (GSVA).^52^ Breast cancer patients from The Cancer Genome Atlas (TCGA) databases with a higher ES score of the LAIT versus GC upregulated gene set in the CD8^+^ and CD4^+^ T cells exhibited prolonged overall survival compared to the patients with lower ES score of such gene set expression (Figure 6C,E). This result was also consistent with that when using upregulated genes from LAIT versus the other three groups combined (LAIT vs CTRL+PTT+GC) for both CD8^+^ and CD4^+^ T cells (Figure 6D,F), indicating that LAIT upregulated gene sets may provide beneficial effect for breast cancer patient prognosis and survival extension.

On the other hand, the LAIT versus GC derived downregulated genes in CD8^+^ T (Figure S6C) and CD4^+^ T (Figure S6E) cells did not affect patient survival. This was also consistent with the results in LAIT versus CTRL+PTT+GC derived downregulated genes (Figure S6D,F). Taken together, we provide predictive evidence that LAIT has a greater potential to prolong patient survival by driving CD8^+^ and CD4^+^ T cell activation and favourable gene signatures that correlate with greater overall survival of breast cancer patients.

## 3 DISCUSSION

Our previous work demonstrated that LAIT had great promise as an effective cancer immunotherapy.^12^, ^14^, ^19^, ^20^, ^21^, ^53^ Recently, we reported that LAIT enhanced antitumor B cell activity by upregulating antigen presentation and interferon signatures, suggesting that the numerous B cells within MMTV‐PyMT tumours may enhance T cell activity.^34^ In this work, we systematically dissected the heterogeneity of T cell populations within the TME (Figures 1 and 2) and the transcriptomic changes responding to PTT, GC, and LAIT at a single‐cell resolution (Figures 2, 3, 4, 5). We discovered that LAIT primarily increased the proportions of activated CD8^+^ and CD4^+^ T cells (Figure 3). LAIT induced gene expression and enriched pathways involved in TCR co‐stimulation, type I IFN response, and a series of antitumor cytokines, including *Ifng*, *Tnf*, *Il1*, and *Il17* in CD8^+^ and CD4^+^ T cells (Figures 3, 4, 5).

We also found that LAIT upregulated the expression of tumour killing molecules *Prf1*, *Gzmb*, *Ifng*, *Tnf*, and *Tnfsf10* (TRAIL) in exhausted and effector memory CD8^+^ and CD4^+^ T cells. The induction of immune checkpoints of *Pdcd1* and *Ctla4* by LAIT (Figures 4F and 5F) supports the notion of T cell activation. More importantly, LAIT‐specific upregulated genes in CD8^+^ and CD4^+^ T cells positively correlated with extended breast cancer patient survival by GSVA analysis of TCGA data (Figure 6 and Figure S6). This suggests that LAIT can alter the TME in such a way that it improves patient outcomes and therapeutic responses to multimodal cancer treatment regimens.

Our results highlight the importance of combining ablation and GC. Tumour ablation utilizes physical means, such as heat, electricity, and radiation, to destroy a tumour locally. Multiple ablation modalities have been developed over the years, such as laser ablation (photothermal therapy, PTT),^54^ radiofrequency ablation (RFA),^55^ microwave ablation (MWA),^56^ high intensity focused ultrasound (HIFU),^57^ cryoablation,^58^ irreversible electroporation (IRE),^59^ and stereotactic body radiation (SBRT).^60^ While ablations can effectively destroy ablated tumours and induce antitumor immune responses, they seldomly achieve long‐term tumour control, particularly against metastatic tumours because the immune stimulation/activation is inconsistent among patients and tumour types with ablation alone. Combining ablation with immune stimulation using a potent immunostimulant, such as GC, can significantly improve therapeutic outcomes, with long‐term, more consistent, tumour‐specific immune responses. As shown in this study, PTT plus GC drives and activates T cell phenotype and it has the potential to generate sustained antitumor immunity.

Clinically, the key to successful tumour treatment depends on the effective reversal of the immunosuppressive TME. Low numbers of tumour‐infiltrating T cells and high numbers of immunosuppressing cells or immature myeloid cells leads to a poor prognosis, treatment resistance, and low overall survival rates of cancer patients.^61^, ^62^, ^63^, ^64^, ^65^, ^66^ Specifically, the suppressive TME poses a detrimental effect on ICI. Based on the TMEs, cancer patients who fail to respond to ICI fall into three categories: Primary resisters, Adaptive resisters, and Acquired resisters.^5^ All the treatment resistance can be attributed mainly to the insufficient T cell responses in the TME.^5^, ^6^

Our previous work demonstrated that LAIT can globally alter the TME and establish a proinflammatory phenotype in tumour‐infiltrating B cells.^34^ Moreover, in this study we have demonstrated that LAIT enhances T cell activation and cytokine production within the TME. Essentially, the LAIT‐induced T cell responses have the potential to overcome the suppressive TMEs of the reported ICI resisters. Based on our accumulated data, we hypothesize that LAIT can overcome the TME resistance mechanisms and synergize with ICI to enhance tumour regression of both treated tumours and untreated metastases.

## 4 MATERIALS AND METHODS

### 4.1 Mice and study approval

Female FVB/NJ wild‐type (stock #001800) and FVB/N transgenic MMTV‐PyMT (stock #002374) mice were purchased from Jackson Laboratories and housed according to institutional guidelines at the University of Oklahoma Health Sciences Center according to the descriptions in our previous work.^67^ Mice handling and experimental procedures were performed in accordance with the University of Oklahoma Health Sciences Center (OUHSC) and Oklahoma Medical Research Foundation (OMRF) Institutional Animal Care and Use Committee (IACUC) regulations and approved protocols (Protocol# 17‐032‐HCL).

### 4.2 Syngeneic tumour cell transplantation

MMTV‐PyMT murine breast tumour organoids were isolated from FVB/N‐Tg (MMTV‐PyVT) 634Mul/J mice as previously described.^33^ The procedures of syngeneic tumour cell transplantation, including cell incubation, washing, resuspension, and injection into mammary fat pad were conducted according to the previously described methods.^67^

### 4.3 Treatment of mouse tumours

Tumour‐bearing mice were treated according to the methods previously described.^67^ When tumours reached 0.5 cm^3^, the tumour site was shaved and mice received one of four treatments: CTRL (without treatment), GC alone, PTT alone, and LAIT (PTT+GC). The mice in GC group received an intratumoural injection of 1% GC in 0.1  ml solution (Immunophotonics, Inc.). For mice in PTT group, the laser parameters were selected based on our previous studies.^53^, ^68^, ^69^, ^70^, ^71^ The tumour was treated by an 805‐nm laser (AngioDynamics, Latham, NY) with a power density of 1 W/cm^2^ for 10 min, using an optical fibre with a diffusion lens (Pioneer Optics, Bloomfield, CT) to deliver uniform light distribution on the treatment surface. For the mice in LAIT group, the tumour was treated by the laser (1 W/cm^2^ for 10 min), followed by an intratumoural injection of GC (0.1 ml at 1%). Ten days after treatment, tumours were collected from selected mice in each group. The remaining mice were observed for up to 60 days or when the tumours reached a size of 2.5 cm^3^ or reached ethical endpoints.

### 4.4 In vivo depletion of T cells

Once tumours reached 0.4 cm^3^, 400 μg of anti‐CD4, ‐CD8, or isotype antibody (BioXcell) was injected i.p. Twenty‐four hours after antibody injection, tumours were treated with LAIT or left untreated. Three days after LAIT treatment, mice were injected with 200 μg antibody, i.p. and maintained on this dose biweekly until tumours reached a size of 2.5 cm^3^ or reached ethical endpoints.

### 4.5 Tumour harvest

Tumour tissues were isolated, minced, and digested with Collagenase IV and DNase I at 37°C for 20–30 min in RPMI. After enzymatic digestion, immune cells were enriched using lymphocyte separation medium (Corning). The enriched cells were then used for flow cytometry analysis.

### 4.6 Fluorescence‐activated cell sorting (FACS) and conventional flow cytometry

All samples were run on the LSR II and data were analysed using FlowJo v10.6.1 (BD Biosciences, San Jose, CA). Briefly cells were stained on ice for 15–20 min with the viability dye Ghost Dye BV510 (Tonbo Bioscience, cat# 13–0870) and the following antibodies: CD45 (Biolegend, cat#103134), CD8 (Tonbo Bioscience, cat# 35–0081), CD44 (Tonbo Bioscience, cat# 65–0441), CD4 (Biolegend, cat# 100456), CD28 (Biolegend, cat# 102104), CD62L (Tonbo Bioscience, cat#60‐0621), CD3 (Tonbo Bioscience, cat#80‐0032), CD69 (Biolegend, cat#104530), CCR7 (Biolegend, cat# 120124), CD25 (Biolegend, cat#102012), Foxp3 (Invitrogen, cat#12‐5773‐82), and CD103 (Biolegend, cat# 121426). Cells were subsequently washed and fixed using the FoxP3 Transcription Factor staining buffer set (ThermoFisher Scientific, cat#00‐5523‐00). The cells used for scRNAseq were depleted of EpCAM^+^ cells using the EasySep Mouse Streptavidin RapidSpheres Isolation Kit (Stem Cell, cat#19860) and then sorted using the MoFlo sorting system based on the expression of CD45 (Biolegend, cat#103112) and viability (Ghost dye BV510, Tonbo Bioscience, cat# 13–0870).

### 4.7 Sample preparation and data processing for single‐cell RNA sequencing

Tumour sample processing, tumour‐infiltrating immune cell preparation, and single‐cell RNA sequencing barcoding and library generation were performed according to our previous work.^67^ Briefly, FACS sorted live CD45^+^ tumour‐infiltrating immune cells were encapsulated into droplets via a 10× Genomics platform according to the manufacturer's instructions. Paired‐end RNA‐seq was performed via an Illumina NovaSeq 6000 sequencing system. The Linux command *cellranger count* from *Cell Ranger* pipeline was used to process sample‐specific *FASTQ* files into feature‐barcode matrices. *Seurat* (v3.2) was used to process and integrate above scRNAseq datasets from different treatment groups.^38^, ^67^, ^72^, ^73^ For quality control, genes that were expressed in less than 5 cells or cells that expressed less than 800 and more than 6000 genes were excluded. Also, cells with a mitochondria percentage over 10% were filtered out. Most variable genes were identified using the *FindVariableFeatures* function by setting feature numbers as 2000. Principal component analysis (PCA) was performed using the first 30 principal components (PCs). Clustering was performed using *FindClusters* that implements a shared nearest neighbour (SNN) modularity optimization‐based clustering algorithm with resolution 0.5 for default analysis. Lymphoid compartment cells were subset and re‐clustered with resolution 0.7 to generate its UMAP plot. The accession number for the scRNAseq data reported in this paper is GEO: GSE150675.^67^ Code for such an analysis can be available upon request.

### 4.8 Differential gene expression (DGE) and functional enrichment analysis

Treatments of PTT, GC, and LAIT (PTT+GC) were compared with CTRL, respectively. *FindMarkers* function in *Seurat* R package was used to obtain differential gene expression for each comparison with threshold of Log fold change (logFC) set at 0.25 and −0.25 as default.^38^ Top 10 of both upregulated and downregulated differentially expressed genes (DEGs) were labelled on volcano plots visualized by using *ggplot2* in R. *clusterProfiler* R package was adopted for gene functional enrichment analysis by using both over‐representation analysis (ORA) and gene set enrichment analysis (GSEA) methods and gene annotation and pathway databases including GO, KEGG, Reactome, and MsigDB.^49^ Dot plot and network plot were mainly used for ORA and GSEA result visualizations, respectively. GSEA obtained normalized enrichment scores (NES) for pathways which were used for clustering in heatmap.

### 4.9 Overlapping of treatment‐derived DEGs

Treatment‐derived DEGs were overlapped and visualized by using *VennDiagram* R package following with subset of indicated gene sets functional enrichment analysis.^49^ Expression of these subset genes were obtained using *AverageExpression* function in *Seurat* R package^38^ and visualized using circular heatmap.^74^, ^75^

### 4.10 Correlation between clinical survival and expression of given gene set

Gene expression RNAseq and survival data available from GDC TCGA Breast Cancer (BRCA) (20 datasets) were downloaded from UCSC Xena (https://xenabrowser.net/datapages/) and used for assessing the clinical significance of expression of a given gene set.^76^ To evaluate whether there was a relationship between differentially expressed gene set and clinical survival in breast cancer patients, we utilized both LAIT versus GC and LAIT versus CTRL+PTT+GC combined groups to obtain the upregulated and downregulated gene sets and visualized in volcano plot. Gene symbols in mouse were converted to gene symbols in human by using *biomaRt* R package.^77^ These gene sets were taken as the input to get the enrichment score (ES) using gene set variation analysis (GSVA) R package.^52^ Cancer patients were stratified as the “high” group if ES was above the median and as “low” group if ES is below median. Analyses were performed with Kaplan‐Meier estimates and log‐rank tests. Numbers below plots represent numbers of breast cancer patients and survival time are days.

### 4.11 Single‐cell trajectory analysis

*Monocle2* R package was chosen for single‐cell trajectory inference using *DDRTree* algorithm.^46^ The tumour‐infiltrating CD8^+^ and CD4^+^ T cell *Seurat* objects were imported into *Monocle2* and highly variable genes calculated by *differentialGeneTest* function were used for gene ordering and trajectory construction.

### 4.12 Statistical analyses

Statistical analysis was performed with GraphPad Prism and R. Unless indicated otherwise, data were expressed as means ±SD or SEM. One‐way analysis of variance (ANOVA) was used for multiple group comparisons. Proportion test function (“prop.test”) in R was used for cell proportion test. Differences in survival were determined on the basis of Kaplan‐Meier survival analysis. Adjusted *P* values less than or equal to .05 were considered statistically significant (*, *p* ≤ .05; **, *p* ≤ .01).

## CONFLICT OF INTEREST

Wei R. Chen is co‐founder and an unpaid member of the Board of Directors of Immunophotonics, Inc. Tomas Hode and Samuel S. K. Lam declare a conflict of interest as employees with minority ownership stakes of Immunophotonics, Inc., the manufacturer of the proprietary immune stimulant GC. Other authors confirm that there is no known conflict of interest associated with this publication. There was no financial support that influenced the conclusions of this manuscript.

## DATA AND CODE AVAILABILITY

The accession number for the scRNAseq data reported in this paper is GEO: GSE150675 (https://www.ncbi.nlm.nih.gov/geo/query/acc.cgi?acc=GSE150675). Analysis of such data can also be available upon request.

## Supporting information

Supporting Information
