# Supplementary material for: Single‐cell RNA sequencing reveals localized tumour ablation and intratumoural immunostimulant delivery potentiate T cell mediated tumour killing
Source: Clin Transl Med. 2022 Jul 8;12(7):e937. doi: 10.1002/ctm2.937 (PMC9270578; doi:10.1002/ctm2.937)
Supplement: Supplementary file 1 — Supporting Information [file CTM2-12-e937-s001.pdf]

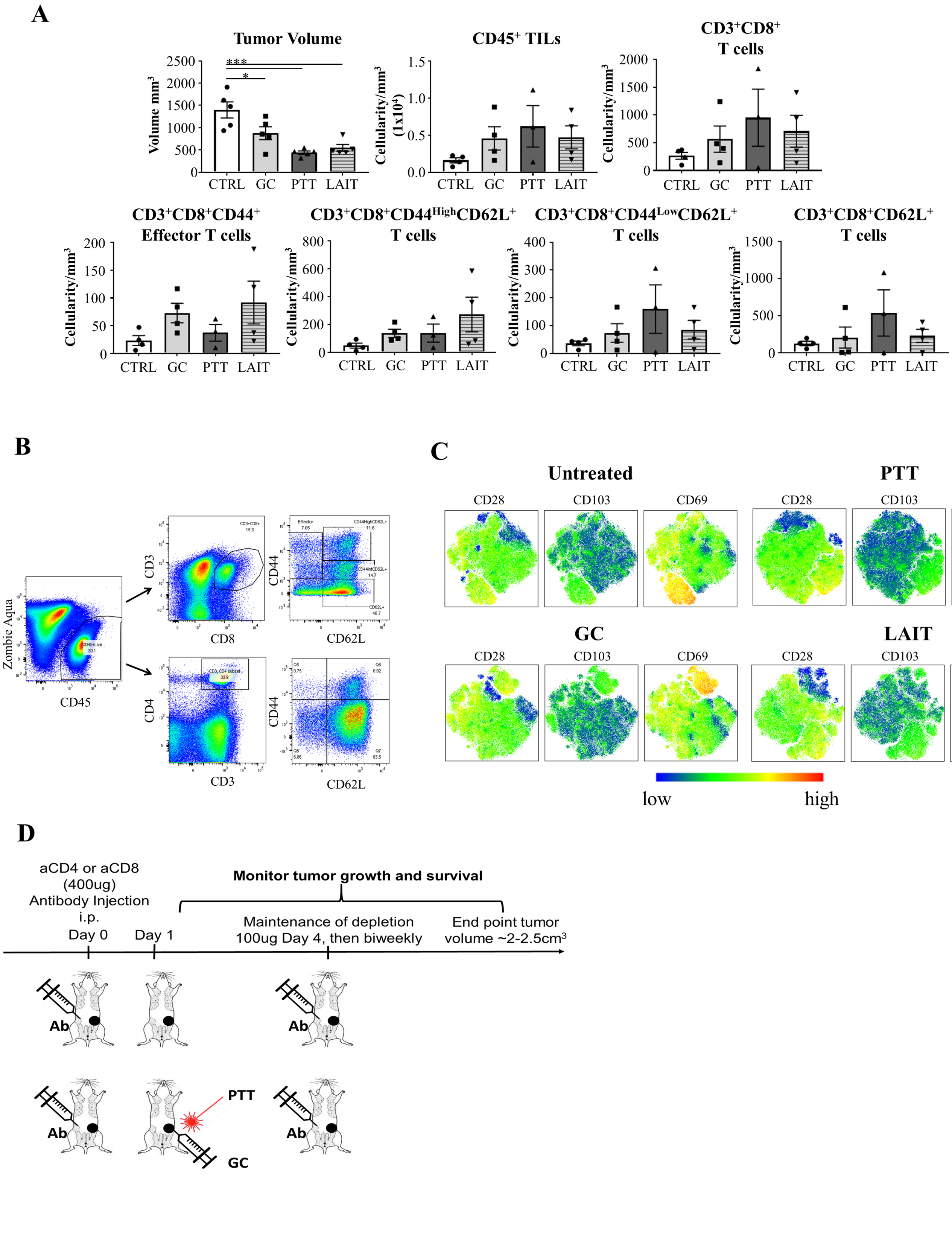
A
Tumor Volume
Volume mm3
CTRL GC PTT LAIT
CD45+ TILs
Cellularity/mm3 (1x10^4)
CD3+CD8+ T cells
Cellularity/mm3
CD3+CD8+CD44+ Effector T cells
CD3+CD8+CD44HighCD62L+ T cells
CD3+CD8+CD44LowCD62L+ T cells
CD3+CD8+CD62L+ T cells
B
Zombie Aqua
CD45
CD3
CD8
CD44
CD62L
CD4
C
Untreated
PTT
GC
LAIT
CD28
CD103
CD69
low
high
D
aCD4 or aCD8 (400ug) Antibody Injection i.p.
Day 0
Day 1
Monitor tumor growth and survival
Maintenance of depletion 100ug Day 4, then biweekly
End point tumor volume ~2-2.5cm3
Ab
PTT
GC

Figure S1.

Figure S1. T cell activation by LAIT (related to Figure 1)

(A) Tumor size, total TILs, and $CD8^+$ T cell cellularity divided by tumor volume.

(B) Gating strategy for $CD4^+$ and $CD8^+$ T cells.

(C) T-SNE plots generated from 3 individual concatenated files in Flowjo of tumor-infiltrating $CD8^+CD3^+$ T cells. T-SNE plots are representative heat maps of the intensity and distribution of CD28, CD103, and CD69 within the $CD8^+CD3^+$ T cells.

(D) Schematic of depletion of $CD4^+$ and $CD8^+$ T cells and LAIT treatment of tumor-bearing mice.

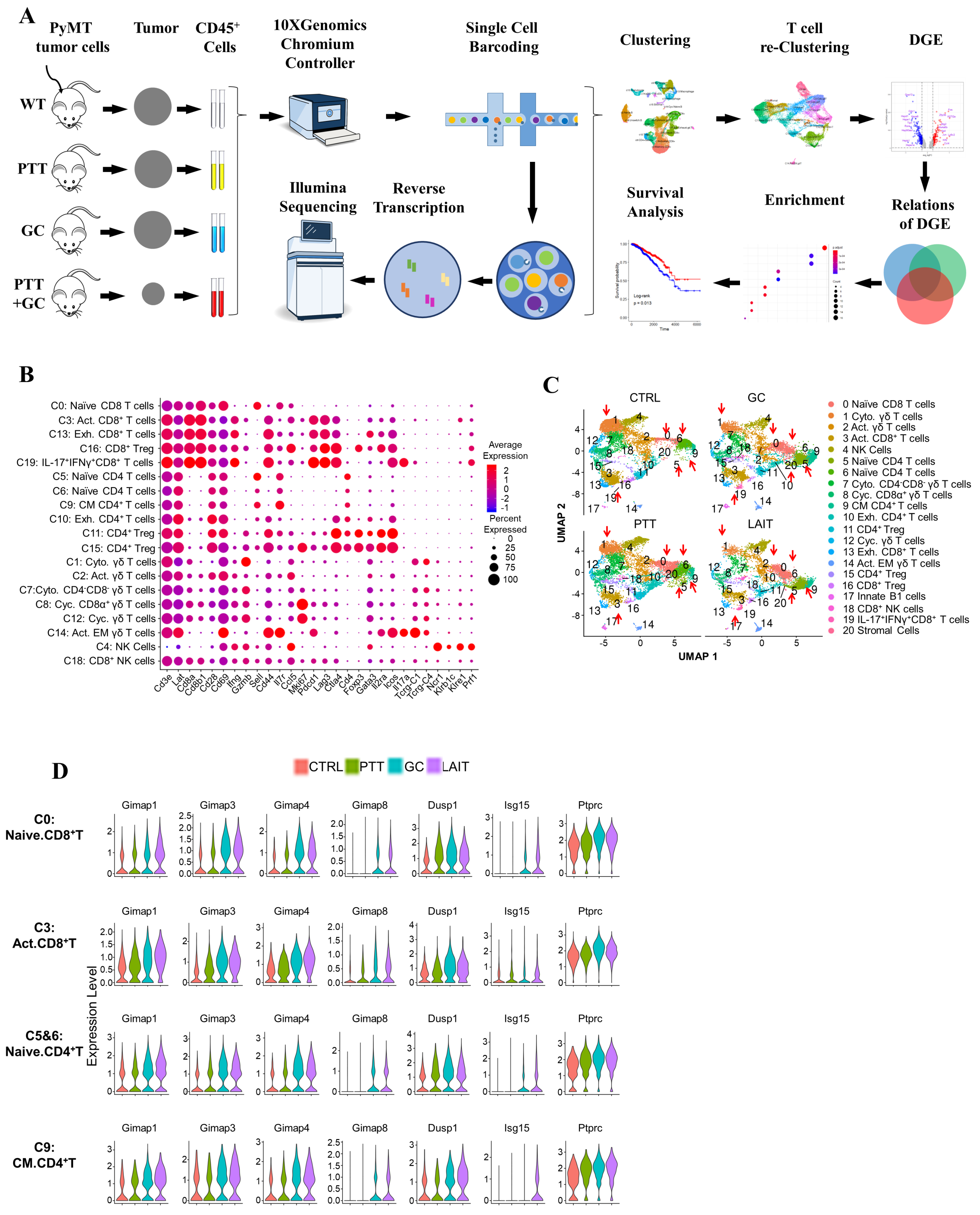
A
PyMT tumor cells
Tumor
CD45+ Cells
10XGenomics Chromium Controller
Single Cell Barcoding
Clustering
T cell re-Clustering
DGE
WT
PTT
GC
PTT +GC
Illumina Sequencing
Reverse Transcription
Survival Analysis
Enrichment
Relations of DGE
B
C0: Naïve CD8 T cells
C3: Act. CD8+ T cells
C13: Exh. CD8+ T cells
C16: CD8+ Treg
C19: IL-17+IFNγ+CD8+ T cells
C5: Naïve CD4 T cells
C6: Naïve CD4 T cells
C9: CM CD4+ T cells
C10: Exh. CD4+ T cells
C11: CD4+ Treg
C15: CD4+ Treg
C1: Cyto. γδ T cells
C2: Act. γδ T cells
C7:Cyto. CD4-CD8- γδ T cells
C8: Cyc. CD8α+ γδ T cells
C12: Cyc. γδ T cells
C14: Act. EM γδ T cells
C4: NK Cells
C18: CD8+ NK cells
Cd3e Lat Cd8a Cd8b1 Cd28 Cd69 Ifng Gzmb Sell Cd44 Il7r Ccl5 Mki67 Pdcd1 Lag3 Ctla4 Cd4 Foxp3 Gata3 Il2ra Icos Il17a Tcrg-C1 Tcrg-C4 Ncr1 Klrb1c Klre1 Prf1
Average Expression
2 1 0 -1 -2
Percent Expressed
0 25 50 75 100
C
CTRL
GC
PTT
LAIT
UMAP 1
UMAP 2
0 Naïve CD8 T cells
1 Cyto. γδ T cells
2 Act. γδ T cells
3 Act. CD8+ T cells
4 NK Cells
5 Naïve CD4 T cells
6 Naïve CD4 T cells
7 Cyto. CD4-CD8- γδ T cells
8 Cyc. CD8α+ γδ T cells
9 CM CD4+ T cells
10 Exh. CD4+ T cells
11 CD4+ Treg
12 Cyc. γδ T cells
13 Exh. CD8+ T cells
14 Act. EM γδ T cells
15 CD4+ Treg
16 CD8+ Treg
17 Innate B1 cells
18 CD8+ NK cells
19 IL-17+IFNγ+CD8+ T cells
20 Stromal Cells
D
CTRL PTT GC LAIT
C0: Naive.CD8+T
C3: Act.CD8+T
C5&6: Naive.CD4+T
C9: CM.CD4+T
Gimap1
Gimap3
Gimap4
Gimap8
Dusp1
Isg15
Ptprc
Expression Level

Figure S2.

Figure S2. Annotation of tumor-infiltrating immune cells using scRNAseq data.

(A) Flowchart for TIL isolation and scRNAseq after different treatments.

(B) Validation of unsupervised clustering using traditional immune cell genes.

(C) UMAP plots of the immune cell atlas from individual treatment groups.

(D) Expression levels of 7 representative DEGs identified in the Venn diagrams from clusters 0, 3, 5/6, and 9.

Figure S3.

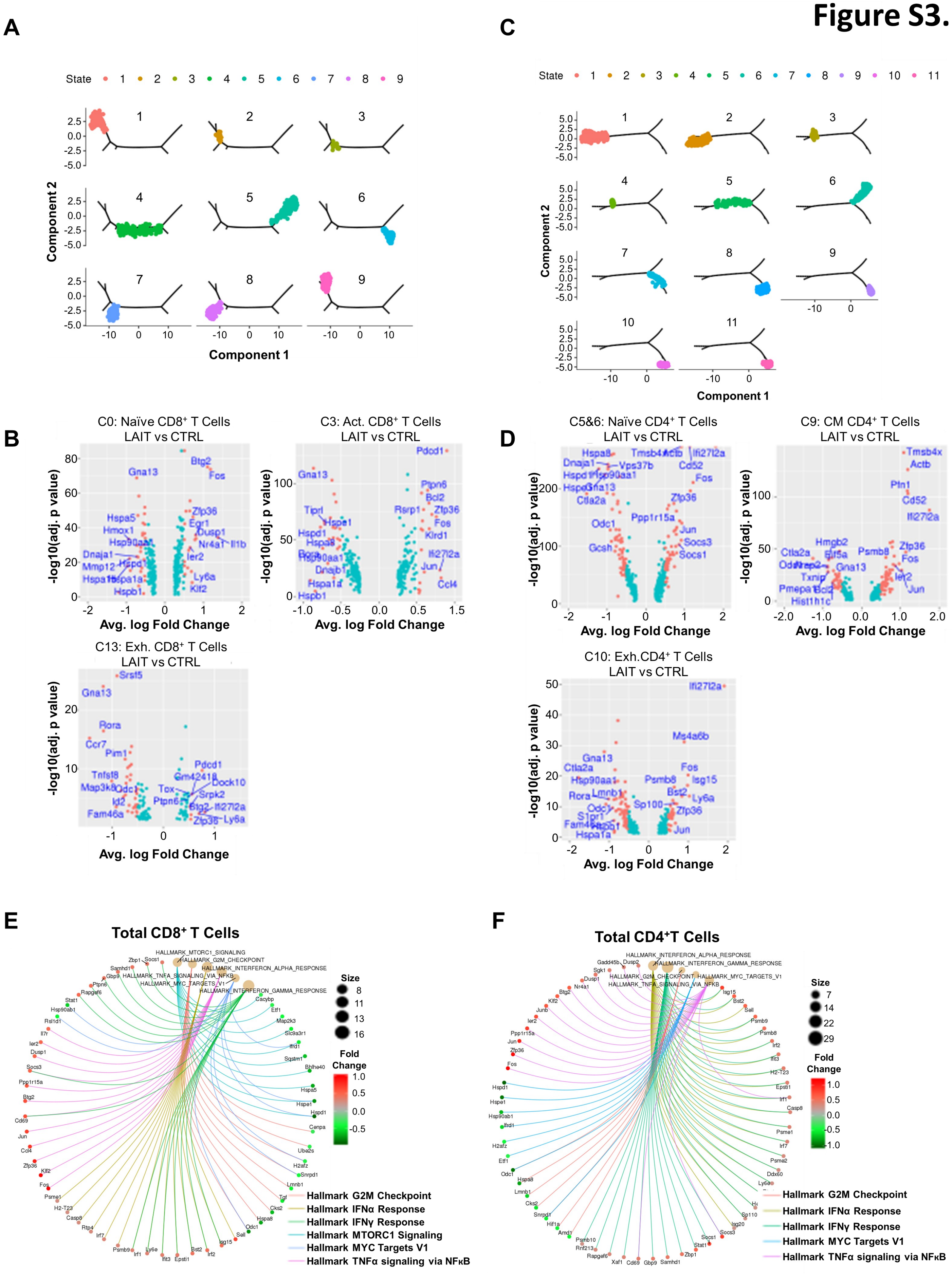
A
State 1 2 3 4 5 6 7 8 9
Component 2
Component 1
B
C0: Naïve CD8⁺ T Cells LAIT vs CTRL
C3: Act. CD8⁺ T Cells LAIT vs CTRL
C13: Exh. CD8⁺ T Cells LAIT vs CTRL
-log10(adj. p value)
Avg. log Fold Change
C
State 1 2 3 4 5 6 7 8 9 10 11
Component 2
Component 1
D
C5&6: Naïve CD4⁺ T Cells LAIT vs CTRL
C9: CM CD4⁺ T Cells LAIT vs CTRL
C10: Exh.CD4⁺ T Cells LAIT vs CTRL
-log10(adj. p value)
Avg. log Fold Change
E
Total CD8⁺ T Cells
Size
Fold Change
Hallmark G2M Checkpoint
Hallmark IFNα Response
Hallmark IFNγ Response
Hallmark MTORC1 Signaling
Hallmark MYC Targets V1
Hallmark TNFα signaling via NFκB
F
Total CD4⁺T Cells
Size
Fold Change
Hallmark G2M Checkpoint
Hallmark IFNα Response
Hallmark IFNγ Response
Hallmark MYC Targets V1
Hallmark TNFα signaling via NFκB

Figure S3. T cells from LAIT-treated tumors are in an activated state (related to Figure 3).

(A) Branched trajectory of $CD8^+$ T cells separated according to each state, generated using monocle2.

(B) Volcano plots of differential gene expression of $CD8^+$ T cells (LAIT versus CTRL) in clusters 0, 3, and 13.

(C) Branched trajectory of $CD4^+$ T cells separated according to each state. States were generated using monocle2.

(D) Volcano plots of differential gene expression of $CD4^+$ T cells (LAIT versus CTRL) in clusters 5/6, 9, and 10.

(E) Pathway enrichment for all $CD8^+$ T cell clusters induced by LAIT using GSEA.

(F) Pathway enrichment for all $CD4^+$ T cell clusters induced by LAIT using GSEA.

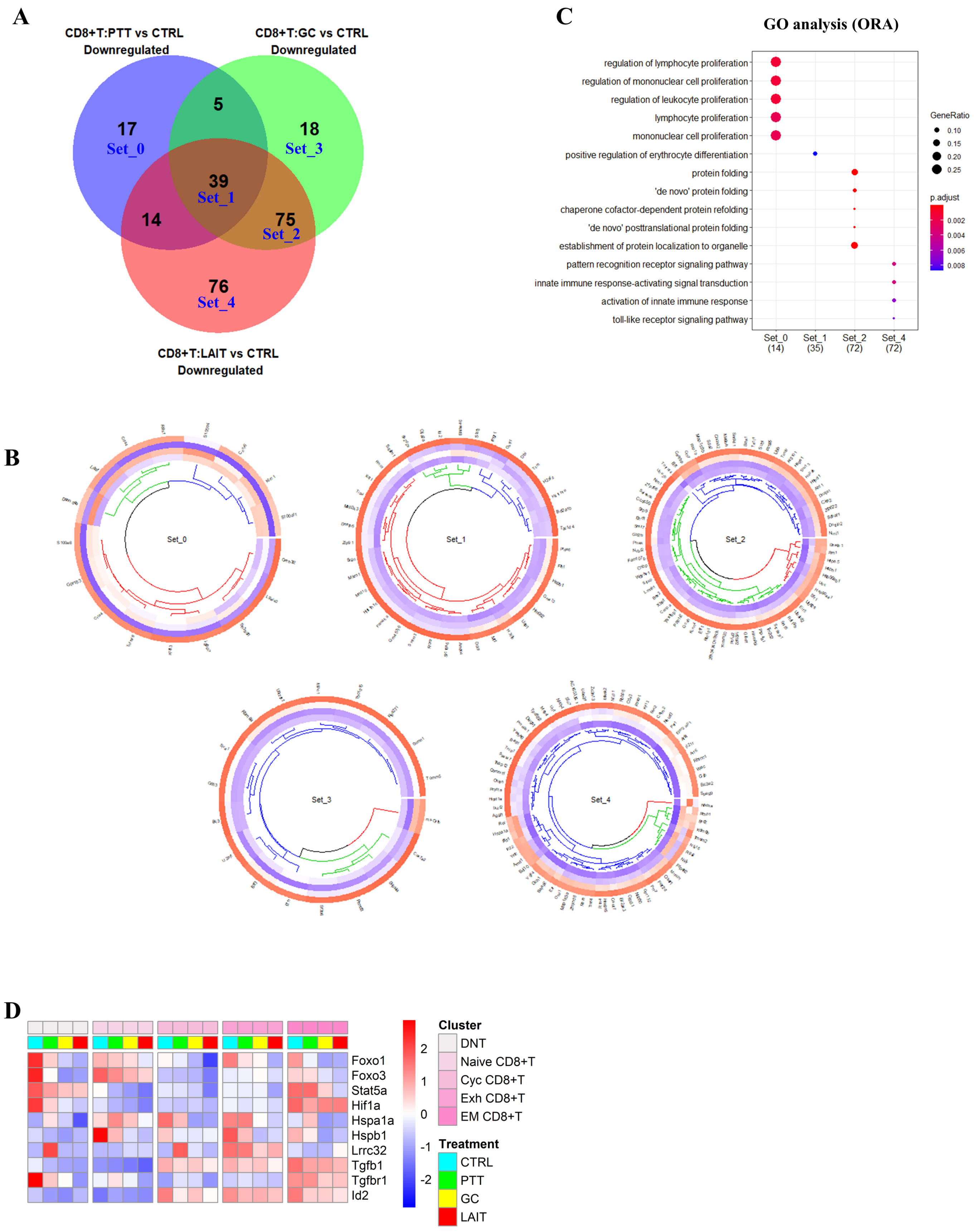
A
CD8+T:PTT vs CTRL Downregulated
CD8+T:GC vs CTRL Downregulated
CD8+T:LAIT vs CTRL Downregulated
17 Set_0
5
18 Set_3
39 Set_1
14
75 Set_2
76 Set_4
C
GO analysis (ORA)
regulation of lymphocyte proliferation
regulation of mononuclear cell proliferation
regulation of leukocyte proliferation
lymphocyte proliferation
mononuclear cell proliferation
positive regulation of erythrocyte differentiation
protein folding
'de novo' protein folding
chaperone cofactor-dependent protein refolding
'de novo' posttranslational protein folding
establishment of protein localization to organelle
pattern recognition receptor signaling pathway
innate immune response-activating signal transduction
activation of innate immune response
toll-like receptor signaling pathway
Set_0 (14)
Set_1 (35)
Set_2 (72)
Set_4 (72)
GeneRatio
0.10
0.15
0.20
0.25
p.adjust
0.002
0.004
0.006
0.008
B
Set_0
Set_1
Set_2
Set_3
Set_4
D
Foxo1
Foxo3
Stat5a
Hif1a
Hspa1a
Hspb1
Lrrc32
Tgfb1
Tgfbr1
Id2
2
1
0
-1
-2
Cluster
DNT
Naive CD8+T
Cyc CD8+T
Exh CD8+T
EM CD8+T
Treatment
CTRL
PTT
GC
LAIT

Figure S4.

Figure S4. Analyses of differential gene expression, pathway enrichment, gene set overlapping and trajectory inference for tumor-infiltrating CD8$^+$ T cell populations.

(A) Venn diagram showing downregulated genes from comparisons of PTT vs CTRL, GC vs CTRL, and LAIT vs CTRL. Five gene sets with large number of overlapping genes, from Set_0 to Set_4, are labeled.

(B) Circular heatmap showing the expression of genes from downregulated Set_0 to Set_4. Heatmap columns for groups of CTRL, PTT, GC and LAIT were arranged from outside to inside. Higher expression was colored in red while lower in blue.

(C) Dot plot for BP of GO analysis of downregulated genes in Set_1 to Set_4.

(D) Heatmap showing the expression of selected genes in each treatment group in each CD8$^+$ T cell subtype.

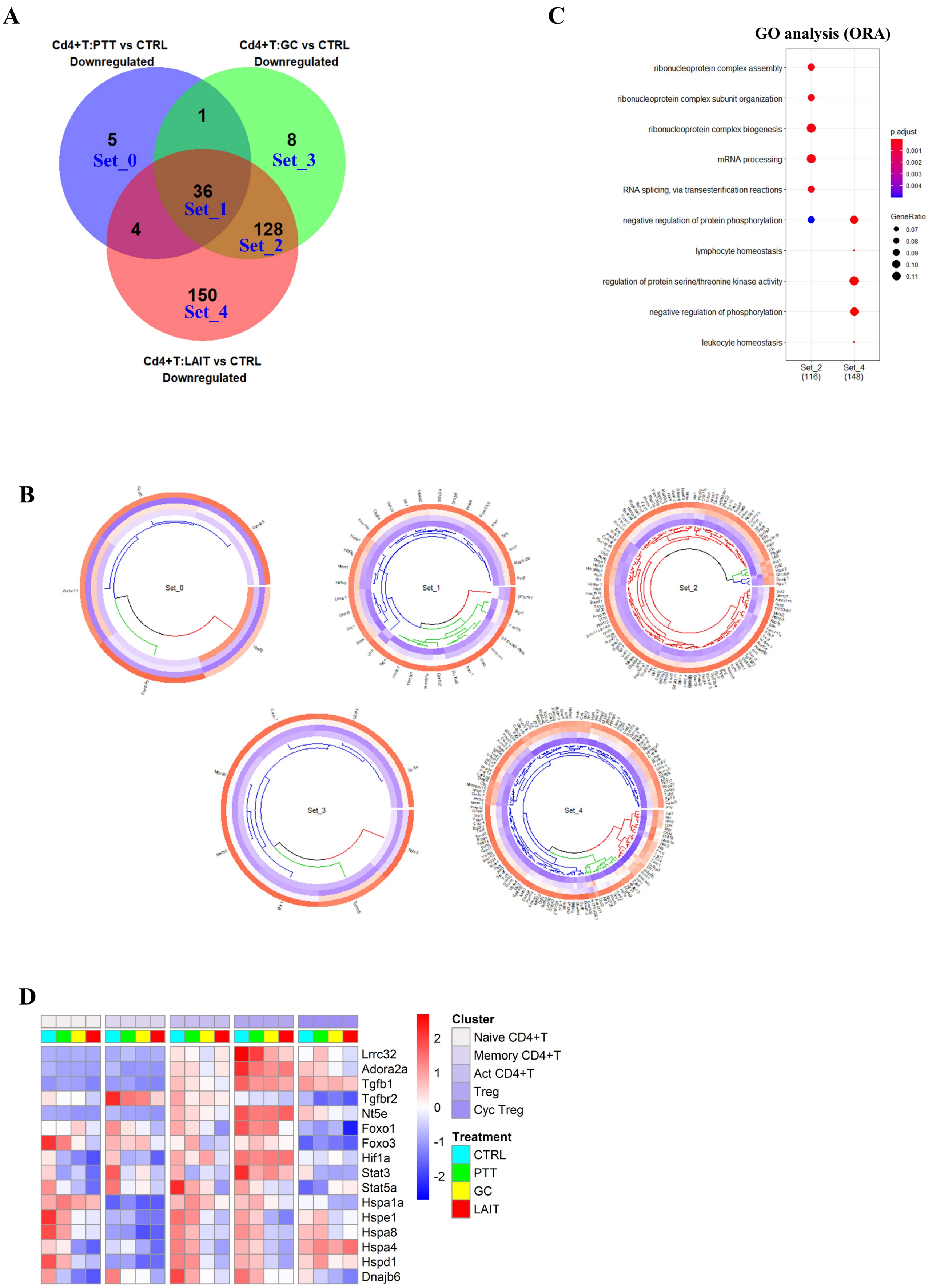
A
Cd4+T:PTT vs CTRL Downregulated
Cd4+T:GC vs CTRL Downregulated
Cd4+T:LAIT vs CTRL Downregulated
5 Set_0
1
8 Set_3
36 Set_1
4
128 Set_2
150 Set_4
C
GO analysis (ORA)
ribonucleoprotein complex assembly
ribonucleoprotein complex subunit organization
ribonucleoprotein complex biogenesis
mRNA processing
RNA splicing, via transesterification reactions
negative regulation of protein phosphorylation
lymphocyte homeostasis
regulation of protein serine/threonine kinase activity
negative regulation of phosphorylation
leukocyte homeostasis
Set_2 (116)
Set_4 (148)
p.adjust
0.001
0.002
0.003
0.004
GeneRatio
0.07
0.08
0.09
0.10
0.11
B
Set_0
Set_1
Set_2
Set_3
Set_4
D
Lrrc32
Adora2a
Tgfb1
Tgfbr2
Nt5e
Foxo1
Foxo3
Hif1a
Stat3
Stat5a
Hspa1a
Hspe1
Hspa8
Hspa4
Hspd1
Dnajb6
2
1
0
-1
-2
Cluster
Naive CD4+T
Memory CD4+T
Act CD4+T
Treg
Cyc Treg
Treatment
CTRL
PTT
GC
LAIT

Figure S5.

Figure S5. Analyses of differential gene expression, pathway enrichment, gene set overlapping and trajectory inference for tumor-infiltrating CD4$^+$ T cell populations.

(A) Venn diagram showing downregulated genes from comparisons of different treatment groups: PTT vs CTRL, GC vs CTRL, and LAIT vs CTRL. Five gene sets with large number of overlapping genes, from Set_0 to Set_4, are labeled.

(B) Circular heatmap showing the expression of genes from downregulated Set_0 to Set_4. Heatmap columns for groups of CTRL, PTT, GC and LAIT were arranged from outside to inside. Higher expression was colored in red while lower in blue.

(C) Dot plot for BP of GO analysis of downregulated genes in Set_1 to Set_4. Only Set_2 and 4 enriched.

(D) Heatmap showing the expression of selected genes in each treatment group in each CD4$^+$ T cell subtype.

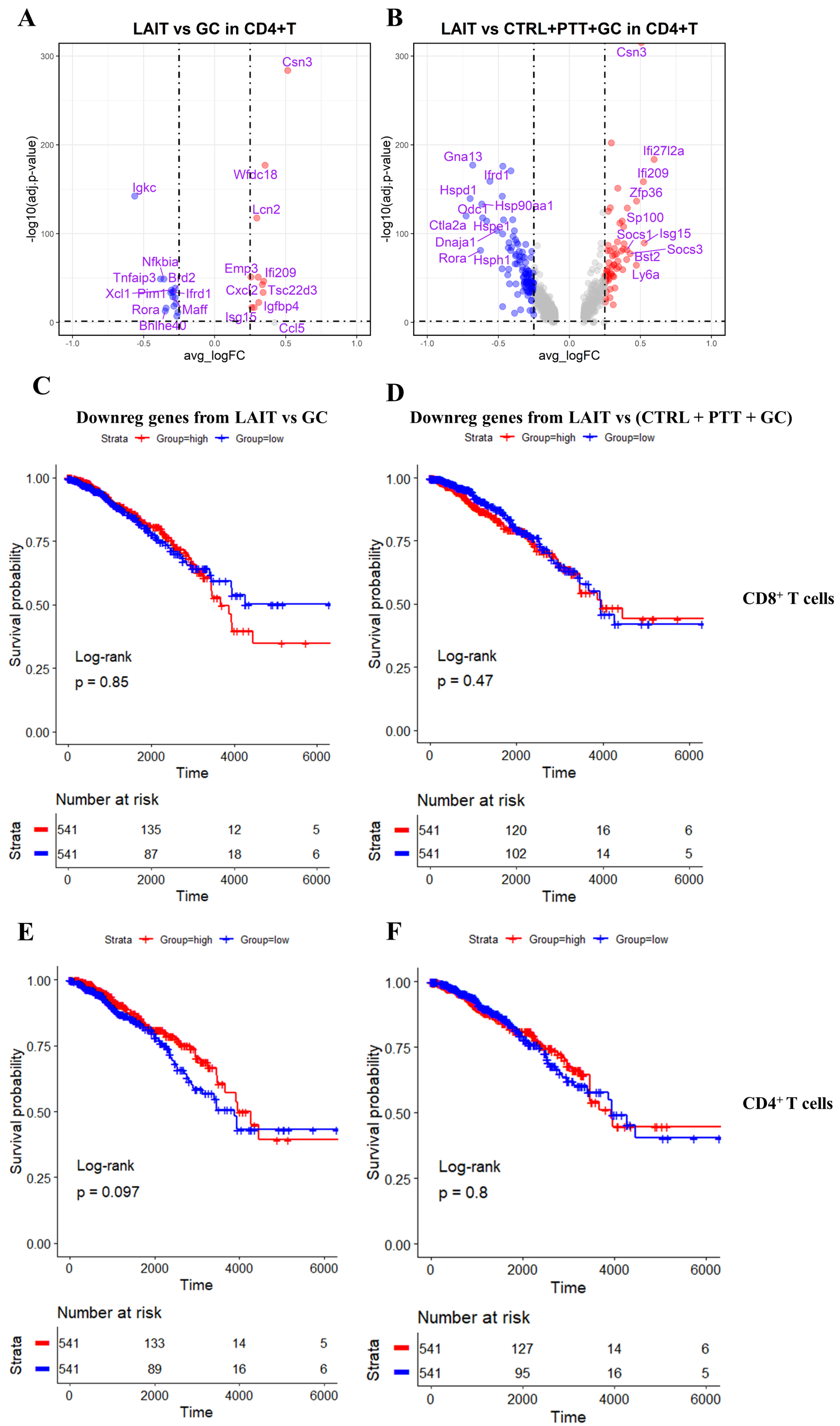

A
LAIT vs GC in CD4+T
B
LAIT vs CTRL+PTT+GC in CD4+T
avg_logFC
-log10(adj.p-value)
C
Downreg genes from LAIT vs GC
D
Downreg genes from LAIT vs (CTRL + PTT + GC)
Strata Group=high Group=low
Log-rank
p = 0.85
Log-rank
p = 0.47
Survival probability
Time
Number at risk
CD8+ T cells
E
F
Log-rank
p = 0.097
Log-rank
p = 0.8
CD4+ T cells

Figure S6.

Figure S6. Association of LAIT specifically downregulated genes in T cell subtypes with breast cancer patient survival.

(A) Volcano plot showing differential gene expression comparing LAIT with GC in $CD4^+$ T cells. Top 10 upregulated (red) and downregulated (blue) genes are labeled.

(B) Volcano plot showing differential gene expression comparing LAIT with other 3 groups (CTRL+PTT+GC) in $CD4^+$ T cells. Top 10 upregulated (red) and downregulated (blue) genes are labeled.

(C) Kaplan–Meier plots showing NO significant difference in survival time (days) between breast cancer patients in groups with "high" and "low" expressions of LAIT vs GC-derived downregulated genes from $CD8^+$ T cells.

(D) Kaplan–Meier plots showing NO significant difference in survival time (days) between breast cancer patients in groups with "high" and "low" expressions of LAIT vs other 3 groups (LAIT vs CTRL+PTT+GC)-derived downregulated genes from $CD8^+$ T cells.

(E) Kaplan–Meier plots showing NO significant difference in survival time (days) between breast cancer patients in groups with "high" and "low" expressions of LAIT vs GC-derived downregulated genes from $CD4^+$ T cells.

(F) Kaplan–Meier plots showing NO significant difference in survival time (days) between breast cancer patients in groups with "high" and "low" expressions of LAIT vs other 3 groups (LAIT vs CTRL+PTT+GC)-derived downregulated genes from $CD4^+$ T cells.

LIT vs CTRL	Column1	LIT vs PTT	Column2	LIT vs GC	Column3
Gene Name	Expression	Gene Name	Expression	Gene Name	Expression
Cnn2	Up	Csn3	Up	Csn3	Up
Ptprcap	Up	Gimap4	Up		
Cd48	Up	Ifi27l2a	Up		
Slc38a2	Up	Gimap3	Up		
Csn3	Up	Hspa5	Down		
Cd52	Up	Hsp90ab1	Down		
Btg2	Up	Tiprl	Down		
Ifi209	Up	Cks2	Down		
Tmsb4x	Up	Nfkbia	Down		
Irf2	Up	Zfp622	Down		
Isg15	Up	Ptp4a1	Down		
Gimap3	Up	Gna13	Down		
Fos	Up	Pim1	Down		
Rsrp1	Up	Ubc	Down		
Actb	Up	H3f3b	Down		
Sept7	Up	Ifrd1	Down		
Dusp1	Up				
Gimap8	Up				
Zfp36	Up				
Gimap1	Up				
Psmb9	Up				
Tbc1d10c	Up				
Sp100	Up				
Gimap4	Up				
Jun	Up				
Ifi27l2a	Up				
Pak2	Up				
Lcp1	Up				
Ptprc	Up				
Evl	Up				
Rsl1d1	Down				
Strap	Down				
Sqstm1	Down				
Fam107b	Down				
Tomm20	Down				
Odc1	Down				
Tiprl	Down				
Pofut2	Down				
Map1lc3b	Down				
Zfp622	Down				
Mkrn1	Down				
Ptp4a1	Down				
Gna13	Down				
Sfr1	Down				
Ubb	Down				
Lmnb1	Down				
Fth1	Down				
Gfod1	Down				
Magoh	Down				
Per1	Down				

Eif5	Down				
Abt1	Down				
Prdx6	Down				
Zfp91	Down				
Hist1h1c	Down				
Tax1bp1	Down				
Srsf5	Down				
Dnajb6	Down				
Ubald2	Down				
Calm2	Down				
Pim1	Down				
Stat3	Down				
Ubc	Down				
H3f3b	Down				
Hnrnpdl	Down				

Supplemental Table 1. Commonly shared DEGs found in selected lymphoid cell populations comparing LAIT vs CTRL, LAIT vs PTT, and LAIT vs GC.

LAIT vs GC, upregulated genes in CD8+T cells	LAIT vs GC, downregulated genes in CD8+T cells	LAIT vs CTRL+PTT+GC, upregulated genes in CD8+T cells	LAIT vs CTRL+PTT+GC, downregulated genes in CD8+T cells
Csn3	R	Csn3	Hspa5
Wfdc18	Igkc	Wfdc18	H3f3b
S100a9	AY036118	S100a9	Gna13
Cxcl2	Kdm6b	Pdcd1	Ifrd1
Krt18	Brd2	Ifi27l2a	Hsp90aa1
Pdcd1	Myh9	Cxcl2	Hspd1
Emp3	Rrbp1	Ms4a6b	Pim1
Ifi27l2a	Nfat5	Sp100	Hspa8
Dapl1	Hspa5	Gimap1	Hsp90ab1
	Csrnp1	Ifi209	Odc1
	Tnfaip3	Shisa5	Srsf5
	Kpna1	Arhgdib	Zfp622
	Ddx3x	Ly6a	Ptp4a1
	Ifrd1	Krt18	Kdm6b
	Nr4a2	Ifi203	Nfkbia
	Nfkbia	Gimap3	Hspe1
	Sytl3	Ms4a6c	Sqstm1
	Dennd4a	Zbp1	Calm2
	Pim1	Slfn2	Cks2
	Thy1	Isg15	Tiprl
	Dusp5	Gimap4	Dnaja1
	Bhlhe40	Stat1	Hsph1
	Lars2	Bst2	Ubald2
	Il2rb	Ly6c2	Fth1
	Rgs2	Sell	Slc9a3r1
	Gm42418	Dapl1	Brd2
	Hspa1b	Klf2	Dnajb6
	Hspa1a	Ccl4	Rhof
	Gm156		Vgll4
			Ubc
			Hspa1a
			Sfr1
			Rrbp1
			Il21r
			Rel
			Tnfaip3
			Ing2
			Rora
			Sdhaf1
			Irf2bp2
			Nfat5
			Bhlhe40
			Pim3
			Csrnp1
			Neurl3
			Tgfb1
			Dnajb1
			Ctla2a
			Phlda1
			Stat3
			Mt1
			Ccr7
			Hspa1b
			Hspb1

Supplemental Table 2. Differential gene expression comparing various groups specifically in CD8+ T cells

LAIT vs GC, upregulated genes in CD4+T cells	LAIT vs GC, downregulated genes in CD4+T cells	LAIT vs CTRL+PTT+GC, upregulated genes in CD4+T cells	LAIT vs CTRL+PTT+GC, downregulated genes in CD4+T cells
Csn3	Igkc	Csn3	Gna13
Wfdc18	Tnfaip3	Wfdc18	Hspa5
Lcn2	Nfkbia	Lcn2	H3f3b
S100a9	Cd74	Ifi27l2a	Ifrd1
Emp3	Xcl1	Ifi209	Tnfaip3
Ifi209	Brd2	Cd52	Hspd1
Cxcl2	Ifrd1	Zfp36	Hsp90aa1
Tsc22d3	Pim1	S100a9	Ctla2a
Igfbp4	Maff	Sp100	Odc1
Isg15	Csrnp1	Actb	Map1lc3b
Ifit3	Kdm6b	Fos	Hsp90ab1
	Vps37b	Ifi206	Hspe1
	Rora	Gimap1	Dnaja1
	Bhlhe40	Emp3	Pim1
	Odc1	Isg15	Hspa8
	AY036118	Ifi203	Cd74
		Shisa5	Srgn
		Ms4a6b	Prdx6
		Ifit3	Hmgb2
		Socs1	Ninj1
		Psmb8	Sub1
		Limd2	Cks2
		Ifit1	Rel
		Socs3	Rora
		Clec2d	Sdhaf1
		Psmb9	Dnajb6
		Xaf1	Fth1
		Bst2	Calm2
		Slfn1	Fam107b
		Jun	Pim3
		Sell	Tgif1
		Irf7	Ptp4a1
		Ly6a	Gfod1
		Ms4a6c	Eif5
		Zbp1	Nfkbia
		Slfn5	Zfp622
		Ifi208	Gdi2
		Klf2	Sh2d2a
		Mitd1	Ubald2
		Mndal	Vgll4
		Trim30a	Hsph1
		Ier2	Prkar1a
		Rtp4	Lmnb1
		Ankrd44	Vps37b
		Btg2	Tax1bp1

		Ifi47	Snrpd1
		Gimap3	Aebp2
		Stat1	Pofut2
		Prkcq	Bbc3
		Rapgef6	Sfr1
		Tmem71	Tiprl
		Ppp1r15a	Amd1
		Gm2682	Gm45716
		H2-T23	Xcl1
		Ripor2	Prkca
		Evl	Il2rb
		Txk	Kdm6b
		Tsc22d3	Gpr132
		Cxcl2	Map1lc3a
		Igfbp4	Calr
		Gbp7	Ube2b
		Gadd45g	Ubl3
		Dapl1	Rcc2
			Neurl3
			Spty2d1
			Ing2
			Avl9
			Hif1a
			Ifngr1
			Etf1
			Ttc14
			Cdk11b
			Sqstm1
			Cish
			Bhlhe40
			Ccr7
			Casp3
			Gcsh
			Got1
			Irf2bp2
			Zfp91
			Bcl10
			Hist1h1c
			Nr4a2
			Tacc1
			H2afz
			Slc3a2
			Chd1
			Nrip1
			Phlda1
			Ubb
			Maff
			Rab8b
			Igkc
			Cdkn1a

			Mt1
			Rgs2
			Klf4
			Bcl2a1b
			Hspa1a
			Tnfsf8
			Fam46a
			Rgs1
			Hspb1
			Dnajb1

Supplemental Table 3. Differential gene expression comparing various groups specifically in CD4+ T cells
